# UBE2O reduces the effectiveness of interferon-α via degradation of IFIT3 in hepatocellular carcinoma

**DOI:** 10.1038/s41419-023-06369-9

**Published:** 2023-12-21

**Authors:** Heng Li, Yao Liu, Can Cheng, Yang Wu, Shu-Hang Liang, Liang Wu, Hong Wang, Cong-yin Tu, Han-Hui Yao, Fan-Zheng Meng, Bo Zhang, Wei Wang, Jia-Bei Wang, Lian-Xin Liu

**Affiliations:** 1 Cheeloo College of Medicine, Shandong University, Jinan, 250002 P. R. China; 2 Department of Hepatobiliary Surgery, The First Affiliated Hospital of USTC, Division of Life Sciences and Medicine, University of Science and Technology of China, Hefei, 230001 China; 3 Anhui Province Key Laboratory of Hepatopancreatobiliary Surgery, Hefei, 230001 China; 4 Anhui Provincial Clinical Research Center for Hepatobiliary Diseases, Hefei, 230001 China; 5 Department of Comprehensive Surgery, The First Affiliated Hospital of University of Science and Technology of China (USTC) West District/Anhui Provincial Cancer Hospital, Hefei, China; 6 Department of Vascular Surgery, The First Affiliated Hospital of USTC, Division of Life Sciences and Medicine, University of Science and Technology of China, Hefei, 230026 China; 7 Department of Gastrointestinal Surgery, The First Affiliated Hospital of USTC, Division of Life Sciences and Medicine, University of Science and Technology of China, Hefei, Anhui 230001 China; 8 Department of Radiation Oncology, The First Affiliated Hospital of USTC, Division of Life Sciences and Medicine, University of Science and Technology of China, Hefei, Anhui 230001 China; 9 Department of Medical Oncology, The First Affiliated Hospital of USTC, Division of Life Sciences and Medicine, University of Science and Technology of China, Hefei, 230001 China

**Keywords:** Predictive markers, Oncogenes

## Abstract

Interferon (IFN) exerts its effects through interferon-stimulated genes (ISGs), but its efficacy is limited by interferon resistance, which can be caused by the ubiquitination of key proteins. UBE2O was initially identified as a promising therapeutic target based on data from the TCGA and iUUCD 2.0 databases. Through the inhibition of UBE2O, interferon α/β signaling and overall interferon signaling were activated. Integrating data from proteomic, mass spectrometry, and survival analyses led to the identification of IFIT3, a mediator of interferon signaling, as a ubiquitination substrate of UBE2O. The results of in vitro and in vivo experiments demonstrated that the knockdown of UBE2O can enhance the efficacy of interferon-α by upregulating IFIT3 expression. K236 was identified as a ubiquitination site in IFIT3, and the results of rescue experiments confirmed that the effect of UBE2O on interferon-α sensitivity is dependent on IFIT3 activity. ATO treatment inhibited UBE2O and increased IFIT3 expression, thereby increasing the effectiveness of interferon-α. In conclusion, these findings suggest that UBE2O worsens the therapeutic effect of interferon-α by targeting IFIT3 for ubiquitination and degradation.

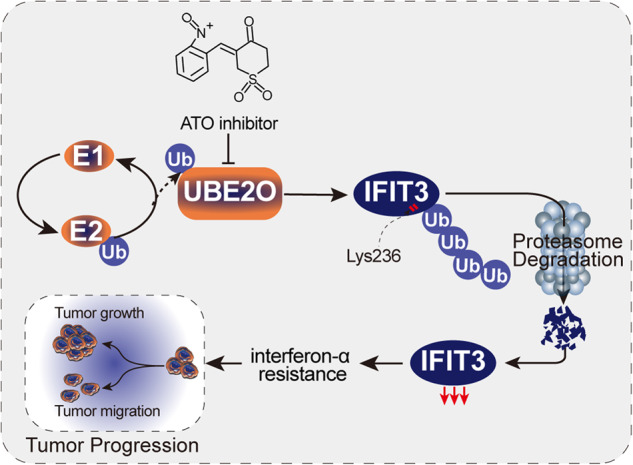

## Introduction

Hepatocellular carcinoma (HCC) accounts for ~80% of all primary liver malignancies; unfortunately, its prognosis is not very good [1]. However, most patients are not diagnosed until the disease is in a late stage and develop resistance to many drugs [2]. Therefore, new and potentially effective strategies such as molecular targeted therapy or immunotherapy are still being explored and developed. Among these strategies, interferon (IFN) is a well-known research focus due to its ability to act as an antitumor agent and enhance the efficacy of immune checkpoint inhibitors [3–5]. Thus, research focused on this field is highly valuable.

Interferons, a specific type of cytokine, play crucial roles in the innate immunological response of humans and were first identified in 1957. Since its approval by the US Food and Medicine Administration (FDA) as the first human cancer immunotherapy drug because of its antiviral and anticancer effects, interferon-α has been widely used to treat viral infections and cancer [6]. The binding of interferon to IFNAR1/2 leads to the expression of interferon-stimulated genes, which enhances innate immunity and inhibits malignant tumor formation [7]. However, resetting tumor cell sensitivity to interferon has become a crucial need in clinical practice due to congenital and acquired factors that cause decreased interferon sensitivity [8, 9]. Studies on interferon resistance in HCC have rarely been reported. IFIT3 is an interferon-induced protein and an inhibitor of cell migration, cell proliferation, apoptosis, and signal transduction. In HCC, high expression of IFIT3 increases the effectiveness of interferon therapy by upregulating the IFN-α effector signaling pathway and response [10]. IFIT3 expression can be used to accurately predict the degree of patient response to interferon therapy [10]. The ubiquitin‒proteasome system plays a crucial role in regulating protein abundance and activity [11]. To date, researchers have identified 40 E2s and between 600 and 1000 E3s [12], which are considered essential determinants of substrate recognition and ubiquitination [13]. Ubiquitination regulates the interferon response and interferon sensitivity by controlling the levels of core proteins in the interferon pathway [14–17]. Thus, a thorough investigation into the ubiquitination of key proteins can allow more precise prediction of the therapeutic effects of interferon and offer guidance for developing strategies to overcome interferon resistance.

Ubiquitin Conjugating Enzyme E2O (UBE2O) has been found to be significantly correlated with the prognosis of hepatocellular carcinoma (HCC), and UBE2O is considered a potential therapeutic target in various types of cancer [13, 18–26]. In HCC, UBE2O promotes lipid metabolic reprogramming and cancer progression by facilitating the ubiquitination of HADHA [27]. However, the relationship between UBE2O and interferon-α efficacy has not been explored to date. Furthermore, at the protein level, the signaling pathway affected by UBE2O remains unclear, as does its association with interferon-induced protein with tetratricopeptide repeats 3 (IFIT3).

Therefore, in this study, we utilized the TCGA LIHC dataset and 807 ubiquitin and ubiquitin-like conjugation-related genes (UUCRGs) obtained from the iUUCD 2.0 database [28] to initially screen for UBE2O substrates. Subsequently, IFIT3 was confirmed to be a novel substrate for UBE2O by proteomic analysis, mass spectrometry, and survival analysis. We employed IP and in vivo ubiquitination assays to identify the ubiquitination sites of IFIT3. In vitro cell functional assays, in vivo xenograft nude mouse models, and rescue assays were employed to evaluate the impact of UBE2O inhibition on interferon-α efficacy. Ultimately, ATO was found to increase IFIT3 protein expression by suppressing UBE2O activity, resulting in improved interferon-α effectiveness.

## Methods

### Public data acquisition

RNA-seq gene expression data (Level 3, FPKM format) of 374 patients with LIHC and 50 normal controls and the clinical information of the patients were downloaded from the TCGA GDC Data Portal (https://portal.gdc.cancer.gov/). Pancancer survival data were downloaded from the UCSC Xena website (https://pancanatlas.xenahubs.net) [28, 29], and the PFI time and survival status of LIHC patients were extracted from these data for additional analysis of differences in PFS between the high-gene-expression group and the corresponding low-gene-expression group. Subsequent bioinformatic analysis and graphical visualization of the results were performed using R software (version 4.1.0) and the corresponding R packages.

### Bioinformatic screening methods for the genes of interest

Six consecutive steps were performed to screen for genes of interest: (1) analysis of differentially expressed genes (DEGs), in which a nonparametric test (unpaired Wilcox test) was used to identify the DEGs in the TCGA LIHC *vs*. the normal control group ( | log2-fold change | >1 & adjusted *p*-value < 0.05 indicated DEGs); (2) merging of common genes from both gene lists, in which the obtained DEGs were merged with the 807 human UUCRGs to identify the overlapping genes; (3) univariate survival analysis, in which the log–rank test and univariate Cox regression analysis were both used for univariate survival analyses based on the merged overlapping genes (log–rank *p* < 0.05 & uniCOX *p* < 0.05 were considered to indicate a statistically significant difference); (4) multivariate survival analysis, in which multivariate Cox regression analysis was performed with the significant genes identified from the above univariate survival analysis (multiCOX *p* < 0.05 was considered to indicate a statistically significant difference); (5) survival ROC analysis, in which ROC analysis was further performed using the SurvivalROC R package based on genes that were identified as statistically significant in the previous step (an AUC >0.7 was used as the valid cutoff value for screening); (6) correlation analysis between gene expression and clinicopathological parameters, in which correlation analyses between the genes and five key clinicopathological parameters (grade, T stage, N stage, M stage, and clinical stage) of the patients were performed (a *p*-value of <0.05 was used as the valid cutoff value for screening).

### Cell culture

293 T, HepG2, Hep-3B, MHCC-97H, HCCLM3, and Huh-7 cells were maintained in DMEM (Gibco) supplemented with 1% penicillin/streptomycin and 10% FBS at 37 °C with 5% CO_2_. SMMC-7721 and WRL68 cells were maintained in RPMI-1640 medium supplemented with 1% penicillin/streptomycin and 10% FBS at 37 °C with 5% CO2. Regular testing was performed to check the source of STR cell lines and for mycoplasma contamination.

### Lentiviral transduction and construction of a stable cell line

When the cell confluence reached 60–70%, the appropriate virus and polyethylenimine were added to the culture. After 8–12 h, the standard medium was replaced, and puromycin (1–5 µg/ml) was added 48 h after infection. When all cells in the control group had died, puromycin was removed, and the surviving cells were expanded and saved for future research.

### RNA extraction and ouantitative RT‒PCR

Total RNA was extracted using the RNeasy Mini Kit (Qiagen). Then, the PrimeScriptTM RT reagent kit (TaKaRa) was used to synthesize cDNA. cDNA was amplified by a SYBR Premix Ex Taq II reagent kit (TaKaRa) and an ABI StepOne Plus instrument (Applied Biosystems).

### In vivo ubiquitination assay

Plasmids containing different tags were transfected into 293 T cells. After 48 h, the proteasome inhibitor MG132 was added, and the cells were incubated for 6 h. Cells were collected for lysis, cellular debris was removed, and the cells were incubated with protein A/G magnetic beads overnight. Ubiquitinated IFIT3 was detected by immunoblotting with an anti-Myc antibody.

### CCK8 Assay

A CCK-8 assay was used to assess the cell proliferation ability of the experimental and control groups. The experimental group was treated with liver tumor cells by adding interferon-α (Kaiyinyisheng, Beijing, China, 1000 U/ml) to the culture medium, while the control group was treated with an equal amount of saline. The cells were then incubated at 37 °C in 5% CO_2_ for 3 days. Subsequently, the above treated cells were seeded in 96-well plates at 2000 cells per well. On the day of the assay, 90 μl of medium and 10 μl of CCK-8 solution were added to each well and then incubated at 37 °C for 2 h. Finally, the OD value of each well was measured at 450 nm.

### Wound healing assay

The indicated cells were treated with human interferon-α (Kaiyinyisheng, Beijing, China) at concentrations ranging from 1000 to 6000 U/ml for a duration of 48–72 h. The cells were washed with PBS, detached, and counted. Approximately 300,000 cells were added to each well of a 6-well plate. After the cells adhered completely to the wall, straight lines were scratched using a ruler and either a 10 µl or 200 µl pipette tip. The cells were then cultured in serum-free medium for 48 h before being photographed.

### Migration assay

The indicated cells were stimulated with 1000–6000 U/ml human interferon-α (Kaiyinyisheng, Beijing, China) for 48–72 h. The cells were serum starved for 12–24 h, detached, and adjusted to a concentration of 2 × 10^5^ cells/ml. A total of 2 × 10^4^ cells were seeded onto the membrane in the upper compartment of the Transwell chamber and allowed to grow for 48 h. The compartment was then removed, and the upper surface of the membrane was gently swabbed before the cells were fixed with methanol for 30 min. Finally, the cells were stained with a solution containing 0.1% crystal violet for a period of 20 min.

### Colony formation assay

The indicated cells were stimulated with 1000–6000 U/ml human interferon-α (Kaiyinyisheng, Beijing, China) for 48-72 h. Then, 1000 cells were seeded in each well of a 6-well plate. After incubation for 14 days, the medium was aspirated, and the cells were washed with PBS, fixed with 4% paraformaldehyde, stained with crystal violet, and imaged.

### Western blot analysis

Briefly, protein samples were prepared and subjected to electrophoresis using a voltage gradient of 80-120 V until the bromophenol blue reached the bottom of the gel. Membrane transfer was performed at a constant current of 280 mA for 90 min in an ice bath using an assembled transfer device. Next, the membrane was blocked with skim milk (5%-8%) for 1 h before incubation with a primary antibody overnight at 4 °C and subsequent incubation with an appropriate secondary antibody for 40–60 min.

### Co-IP assay

When the cell confluence reached 80-90%, the cells were washed, detached and collected. After centrifugation, the supernatant was mixed with the appropriate amount of antibody and incubated at 4 °C overnight. Then, 20 µl of beads was added to each sample, and the sample was mixed at 4 °C for 2 h. The beads were washed three times, resuspended in protein lysis buffer and added to the same volume of 2 × loading buffer, and proteins were denatured by boiling for 10 min. Finally, the target protein was detected by Western blotting with the corresponding antibody.

### Cellular immunofluorescence

The experimental procedure primarily involves cell fixation, permeabilization, blocking, incubation with primary antibodies, washing steps, secondary antibody incubation, nuclear staining, sealing and imaging. In brief, initially, the cells were fixed and permeabilized to enhance antibody binding. Following blocking, a specific primary antibody targeting the molecule of interest was introduced and thoroughly washed to eliminate any unbound antibodies. Subsequently, fluorescently labeled secondary antibodies were employed, and after another round of washing steps, DAPI staining was performed on the cells. Finally, the cells were mounted onto slides for observation using a fluorescence microscope.

### Cycloheximide (CHX) assay

The final concentration of CHX was adjusted to 80 µg/ml. Subsequently, the cells were treated with CHX at this concentration, and the experiment was terminated at the established time point. The harvested cells were then used for protein sample preparation, followed by Western blot analysis for detection of target proteins. Finally, quantitative analysis was performed using ImageJ.

### CKSAAP

CKSAAP (http://systbio.cau.edu.cn/cksaap_ubsite/) [30, 31], a predictor based on a support vector machine, has been proven to outperform other predictors in determining ubiquitination sites.

### GPS-Uber database

GPS-Uber [32], (http://gpsuber.biocuckoo.cn/) was developed as a hybrid learning approach to predict the sites of lysine ubiquitination with high accuracy.

### Establishment of an in vivo xenograft nude mouse model

After establishment of the stable Sh UBE2O cell line, Western blotting was performed to validate the knockdown efficiency. Three million experimental and control cells were subcutaneously transplanted into nude mice. Subcutaneous injection of interferon-α(5 × 10^6^ U/kg recombinant IFNα-2b, daily, Kaiyinyisheng, Beijing, China) or an equal amount of saline, respectively. Subcutaneous tumors were measured at regular intervals after injection.

Nude mice were subcutaneously injected with 3 million HCCLM3 cells. After 14 days, nude mice were treated with ATO (2.5 mg/kg and 5 mg/kg, intraperitoneal injection, daily, Harbin, China) in combination with interferon-α (5 × 106 U/kg, daily) or ATO (2.5 mg/kg and 5 mg/kg) alone. The protocol for the animal experiment complied with the ARRIVE guidelines and was approved by the Animal Research Ethics Committee of The First Affiliated Hospital of USTC.

### Patients and tissue samples

Hepatocellular carcinoma tissues and the corresponding adjacent noncancerous tissues were obtained during routine surgeries conducted between 2019 and 2022 at the First Affiliated Hospital of USTC. Each patient was histopathologically diagnosed according to WHO guidelines, and written informed consent was obtained from all patients after ethical approval of the study from The First Affiliated Hospital of the USTC Research Ethics Committee.

### Statistical analysis

Biochemical and cell culture assays were repeated three times. GraphPad Prism (Version 9.0) was used for statistical analysis to compare data between and among experimental groups. Data are presented as the mean ± SD unless otherwise specified, with each experiment conducted independently at least three times. Ordinary one-way/two-way ANOVA with multiple comparisons testing was used to examine the statistical significance of differences among three independent groups. An unpaired *t*-test was used to examine the statistical significance of differences between two independent groups. Statistical results are listed in the figure legends, with 0.05 considered statistically significant (*p < 0.05; **p < 0.01; ***p < 0.001).

## Results

### UBE2O regulates IFIT3 expression at the translational level

**Fig. 1:**
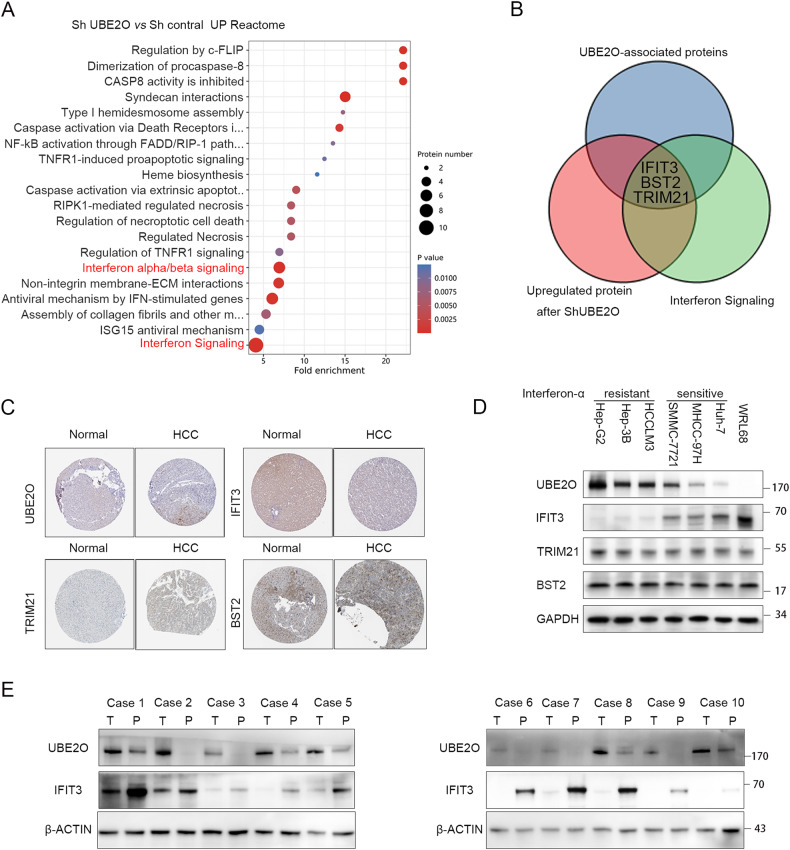
UBE2O negatively regulates IFIT3 expression in HCC. **A** Cells stably transduced with Sh-UBE2O were established using lentiviral transduction, and proteomic analysis was performed with controls. Reactome enrichment analysis was performed with the upregulated differentially expressed proteins. Reactome enrichment analysis was performed with the ReactomePA R package. **B** Immunoprecipitation was conducted in HCCLM3 cells using an anti-UBE2O antibody, and the samples were sent for mass spectrometry analysis to identify proteins bound to UBE2O (indicated by blue circles). Proteins upregulated after UBE2O knockdown are indicated by red circles, while those involved in the interferon pathway are indicated by green circles. The proteins overlapping among these three groups were identified as IFIT3, BST2, and TRIM21. **C** The expression of UBE2O, IFIT3, TRIM21, and BST2 was queried in the HPA database. UBE2O and IFIT3 exhibited opposing expression trends; UBE2O was highly expressed, but IFIT3 was expressed at low levels in HCC tissues, whereas normal liver tissue showed low expression of UBE2O and high expression of IFIT3. Both UBE2O and TRIM21, as well as BST2, displayed similar trends, with high expression observed in HCC tissues but low expression observed in normal liver tissues. **D** In HCC cell lines, UBE2O was highly expressed in tumor cells and expressed at low levels in normal cells. In contrast, IFIT3 was expressed at low levels in tumor cells and highly expressed in normal hepatocytes (*n* = 3). Cell lines with low UBE2O combined with high IFIT3 are interferon-α sensitive, whereas cells with high UBE2O combined with low IFIT3 are interferon-resistant. **E** The protein expression levels of UBE2O and IFIT3 were determined in patients with hepatocellular carcinoma (*n* = 3).

UBE2O was ultimately selected through a comprehensive screening process that involved gene expression analysis, correlation analysis with clinical parameters, and evaluation of patient prognosis (Fig. S1A–F, Tables S1–7). Stably transfected HCCLM3 cells with UBE2O knockdown were generated, and proteomic analysis was conducted to identify the upregulated proteins. The results revealed a significant impact on interferon α/β signaling (*p* < 0.01) and interferon signaling (*p* < 0.01) (Fig. 1A). The Venn diagrams showed that UBE2O bound to (Table S8) and negatively regulated the expression of IFIT3, BST2, and TRIM21 in the interferon signaling pathway (Fig. 1B). Based on the HPA database, UBE2O expression has a negative correlation with IFIT3 expression but a positive correlation with TRIM21 and BST2 expression (Fig. 1C). The expression levels of UBE2O, BST2, TRIM21, and IFIT3 were also measured in HCC cell lines (Fig. 1D). UBE2O was highly expressed in six liver tumor cell lines but had low expression in normal WRL68 cells. Conversely, IFIT3 was expressed most highly in WRL68 cells but had low expression in liver tumor cells. This negative correlation was not observed at the transcriptional level (Fig. S9). Furthermore, we found that cells with high UBE2O expression and concomitant low IFIT3 expression were resistant to interferon-α, while cells with low UBEO2 expression and concomitant high IFIT3 expression were sensitive to interferon-α (Fig. 1D, Fig. S4A). The sensitivity of liver tumor cells to interferon-α was decreased after IFIT3 inhibition (Fig. S4B–E). The expression of TRIM21 and BST2 did not differ significantly among the cell lines. Survival analysis showed that patients with high UBE2O expression had longer overall survival (OS) times, whereas those with low IFIT3 expression demonstrated a more favorable prognosis (Fig. S1E, F, S2A). Patients with high UBE2O expression and low IFIT3 expression had the worst overall survival (OS) and progression-free survival (PFS) outcomes among the patient subgroups (Fig. S2B). There was a negative correlation between UBE2O and IFIT3 protein expression in hepatocellular carcinoma patients (Fig. 1E).

### Inhibition of UBE2O increased interferon-α efficacy

**Fig. 2:**
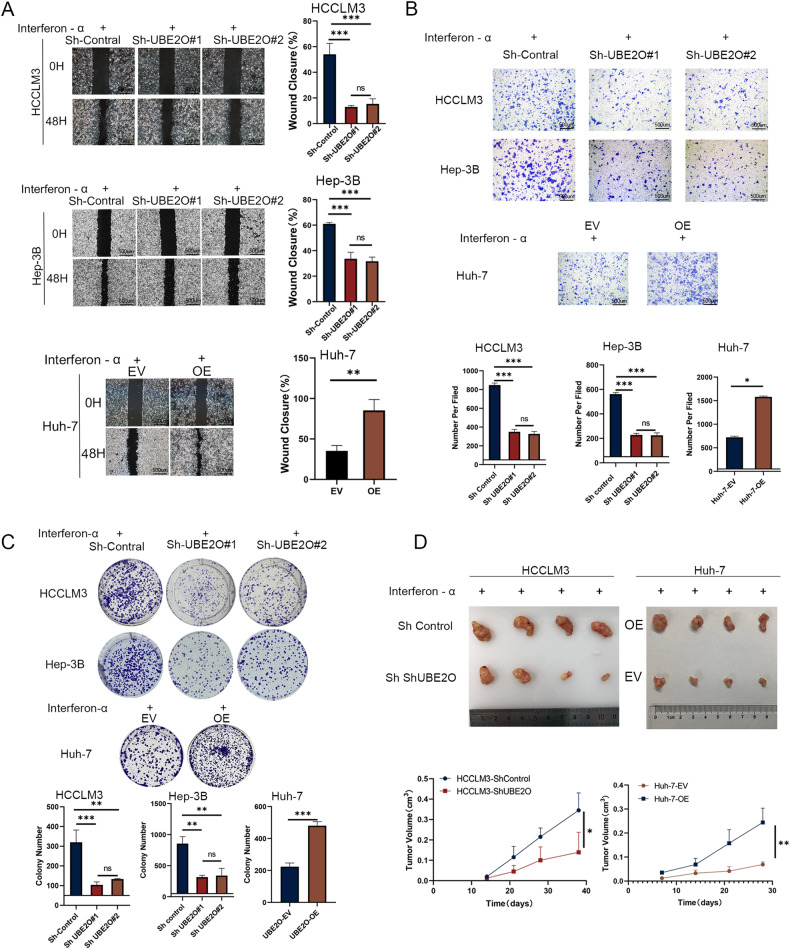
Inhibition of UBE2O can increase the therapeutic effect of interferon-α. After knockdown or overexpression of UBE2O and treatment with equal concentrations of interferon-α, wound healing (**A**) migration (**B**) and colony formation assays (**C**) were conducted to assess changes in HCC cell sensitivity to interferon. The data are expressed as the mean ± SD of three independent experiments. *p < 0.05, **p < 0.01, ***p < 0.001. The pictures are representative of the results of one experiment. Ordinary one-way ANOVA with multiple comparisons testing was used to examine the statistical significance of differences among the three independent groups. Unpaired *t*-test was used for comparisons between two independent groups. **D** Cells from the experimental and control groups were injected subcutaneously into the inguinal region of nude mice, followed by treatment with equal concentrations of interferon-α (5 × 10^6^ U/kg per day). Tumor volumes were calculated every 3 days (*n* = 4 mice/group). The data are shown as the means ± SDs. *p < 0.05, **p < 0.01. Ordinary one-way ANOVA with multiple comparisons testing was used for statistical analysis.

We generated stable cell lines with UBE2O knockdown and overexpression and conducted cell functional assays in vitro. Our results showed that the therapeutic effects of interferon-α were better in the UBE2O inhibition group than in the control group after the same treatment. Knockdown of UBE2O in interferon-resistant cells significantly improved interferon efficacy (Fig. S5A–D). Specifically, we observed significant reductions in the wound healing ability, migration ability, and colony-forming ability of HCCLM3 and Hep-3B cells upon UBE2O knockdown. In contrast, Huh-7 cells exhibited increased sensitivity to interferon-α (Fig. 2A–C) (all *p* < 0.05). The results in the in vivo animal models confirmed that the tumor volume was significantly lower in the UBE2O knockdown group than in the control group after administration of the same interferon treatment regimen (Fig. 2D) (*p* < 0.01).

### IFIT3 is identified as a UBE2O-interacting protein

**Fig. 3:**
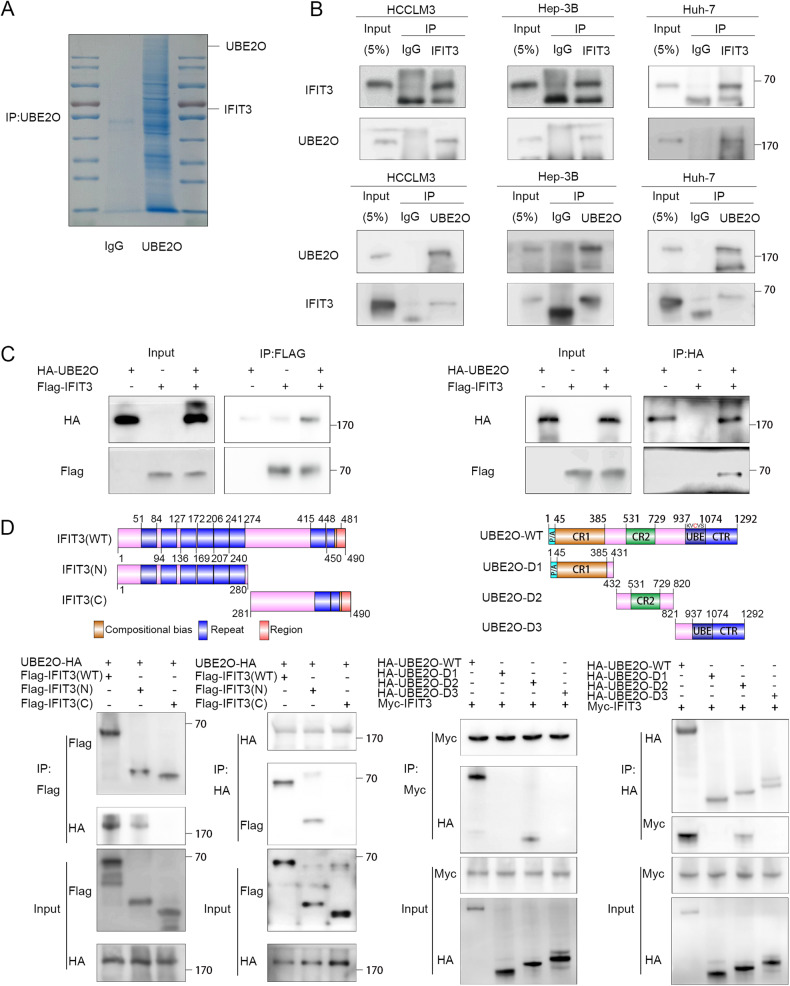
UBE2O interacts with IFIT3. **A** Immunoprecipitation was carried out using anti-rabbit IgG and an anti-UBE2O antibody. Following electrophoresis, the gel strips were stained with Coomassie blue. **B** The indicated cells were lysed in buffer and then subjected to Co-IP analysis with protein A/G magnetic beads and anti-UBE2O or anti-IFIT3 antibodies, followed by Western blot analysis (*n* = 3). **C** 293 T cells were transfected with plasmids containing different tags. After 48 h, the cells were treated with MG132 (10 μM) for 4-6 h. Cells were lysed with buffer and then subjected to Co-IP analysis with protein A/G beads and antibodies specific for the corresponding tags, followed by Western blot analysis (*n* = 3). **D** Truncation mutants of IFIT3 and UBE2O were generated as indicated, and plasmids carrying different tags were transfected into 293 T cells. IP was performed using anti-HA and anti-Myc antibodies, followed by IB with the corresponding antibodies.

To gain a more comprehensive understanding of the correlation between UBE2O and the interferon pathway, we measured the binding of UBE2O to several specific proteins (Fig. S11C). Our findings revealed that only IFIT3 exhibited universal binding to UBE2O. Through our experiments, we were able to confirm this mutual binding relationship between UBE2O and IFIT3 both exogenously and endogenously in HCCLM3, Hep-3B, and Huh-7 cells (Fig. 3A–C, and Fig. S8). We generated truncation mutants of UBE2O and IFIT3 based on the research of Huang [33] and Cao [10] to identify the structural domains of IFIT3 and UBE2O that are responsible for their interaction (Fig. 3D, upper panels). Based on the IP data, their binding is mediated by the N-terminus of IFIT3 and the D2 structural domain of UBE2O (Fig. 3D, and Fig. S11A). In conclusion, these data suggest that UBE2O interacts with IFIT3.

### UBE2O promotes the polyubiquitination and degradation of IFIT3

**Fig. 4:**
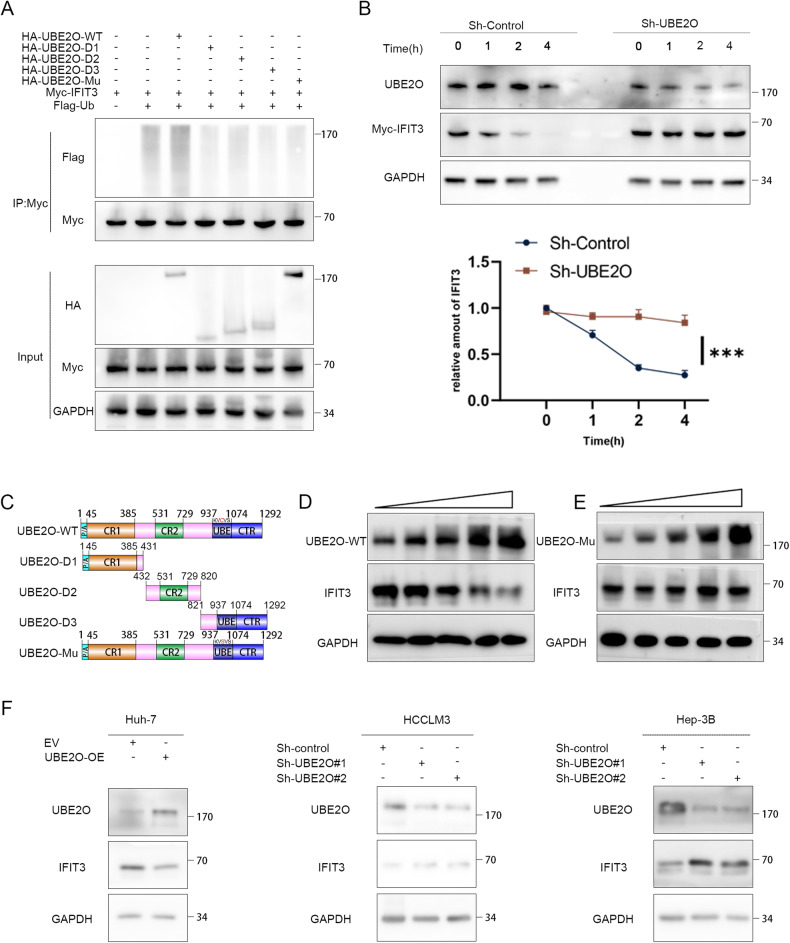
IFIT3 protein stability is regulated by UBE2O. **A** 293 T cells were transfected with the designated plasmids and subsequently exposed to MG132 (10 μM) for a duration of 6 h. The lysed cells were then subjected to coimmunoprecipitation using anti-Myc antibodies, followed by Western blot analysis utilizing anti-Flag antibodies (*n* = 3). **B** Stably transfected cells with UBE2O knockdown were established and validated. The cells were treated with cycloheximide (80 μg/mL), and protein was harvested at designated time points for Western blot analysis. Relative band densities were quantified by ImageJ. The data are expressed as the mean ± SD of three independent experiments. Ordinary one-way ANOVA with multiple comparisons testing was used for statistical analysis. *p < 0.05. **C** Schematic presentation of wild-type UBE2O and the UBE2O mutants. **D** Gradient overexpression of UBE2O-WT (0.5 µg, 1.5 µg, 2.5 µg, 3.5 µg, and 4.5 µg) was performed, and after 48 h of incubation, the cells were lysed to obtain ~400 ml of total cellular protein per dish for subsequent Western blot analysis of IFIT3 protein expression (*n* = 3). **E** Similarly, the amount of the UBE2O-mutant (M3) plasmid transfected was incrementally increased (0.5 µg, 1.5 µg, 2.5 µg, 3.5 µg, 4.5 µg), and the corresponding IFIT3 protein expression levels were measured 48 h posttransfection (*n* = 3). **F** In HCCLM3 and Hep-3B cells, knockdown of UBE2O resulted in an increase in the IFIT3 protein level, but overexpression of UBE2O led to a decrease in the IFIT3 protein level in Huh-7 cells (*n* = 3).

We hypothesized that IFIT3 may be a substrate for ubiquitination by UBE2O, as evidenced by its reduced expression upon CHX treatment and subsequent restoration of its expression by MG132 treatment (Fig. S3A, D). shRNA-mediated knockdown of UBE2O led to upregulation of IFIT3 protein expression in HCCLM3 and Hep-3B cells, while overexpression of UBE2O resulted in decreased levels of IFIT3 in Huh-7 cells (Fig. 4F). Based on Huang’s [20, 33] research, we constructed four UBE2O mutants (Fig. 4C). Wild-type (WT) UBE2O was found to significantly promote the polyubiquitination of IFIT3 compared to the UBE2O mutant (Fig. 4A, Fig. S3C, Fig. S11B). Consistent with this finding, inhibition of UBE2O prolonged the half-life of endogenous IFIT3 (*p* < 0.05) (Fig. 4B, and Fig. S3B). Gradient overexpression of UBE2O resulted in a gradual decrease in the IFIT3 level (Fig. 4D), whereas no significant change in the IFIT3 level was observed after gradient overexpression of any of the UBE2O mutants (Fig. 4E).

### UBE2O enhances ubiquitination of the IFIT3 protein at K236

**Fig. 5:**
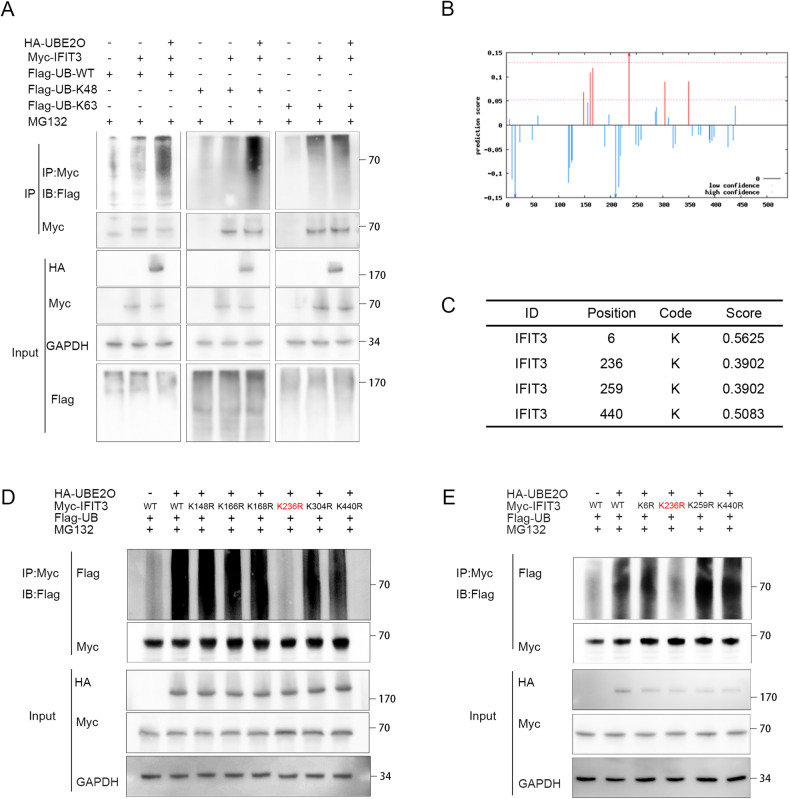
K236 is the site of UBE2O-mediated ubiquitination in IFIT3. **A** Plasmids containing different tags were cotransfected into 293 T cells. Immunoprecipitation using an anti-Myc antibody, followed by immunoblotting (IB) with the corresponding antibodies, was conducted to determine the type of ubiquitination (*n* = 3). **B** Prediction of ubiquitination sites in IFIT3 using the CKSAAP database. **C** Prediction of ubiquitination sites in IFIT3 using the GPS-Uber database. **D** IFIT3 mutants were generated based on prediction results from the CKSAAP database and transfected into 293 T cells for the in vivo ubiquitination assay. Mutation of K236 abolished the ubiquitination-mediated degradation of IFIT3 via UBE2O. **E** Based on the prediction results from the GPS-Uber database, mutations were introduced into IFIT3, and plasmids with the appropriate tags were transfected. IP was performed using an anti-Myc antibody, while IB was carried out using the corresponding antibody. Mutation of K236 abolished the ubiquitination-mediated degradation of IFIT3.

Coimmunoprecipitation assays were performed after transfection with the corresponding plasmids. The results of the ubiquitin-based immunoprecipitation assay revealed that UBE2O facilitated overall polyubiquitination and K48-linked ubiquitination of IFIT3 in 293 T cells but had no impact on K63-linked ubiquitination of IFIT3 (Fig. 5A). We constructed truncations of the IFIT3 gene body and found that UBE2O bound only to the N-terminal end (Fig. 3D), suggesting a possible ubiquitination site in this region. We utilized GPS-Uber (Fig. 5C) and CKSAAP (Fig. 5B) to predict the ubiquitination site in IFIT3 and found that mutation of K236 reversed the degradation resulting from UBE2O-mediated ubiquitination (Fig. 5D, E). The K236 site exhibits high conservation, and the K236R mutation leads to a prolonged half-life of IFIT3(Fig. S3E, F). The mutation of IFIT3-WT to IFIT3-236R reversed the elevation of interferon-α sensitivity (Fig. S6A–D).

### IFIT3 reverses UBE2O knockdown-mediated interferon-α treatment potentiation

**Fig. 6:**
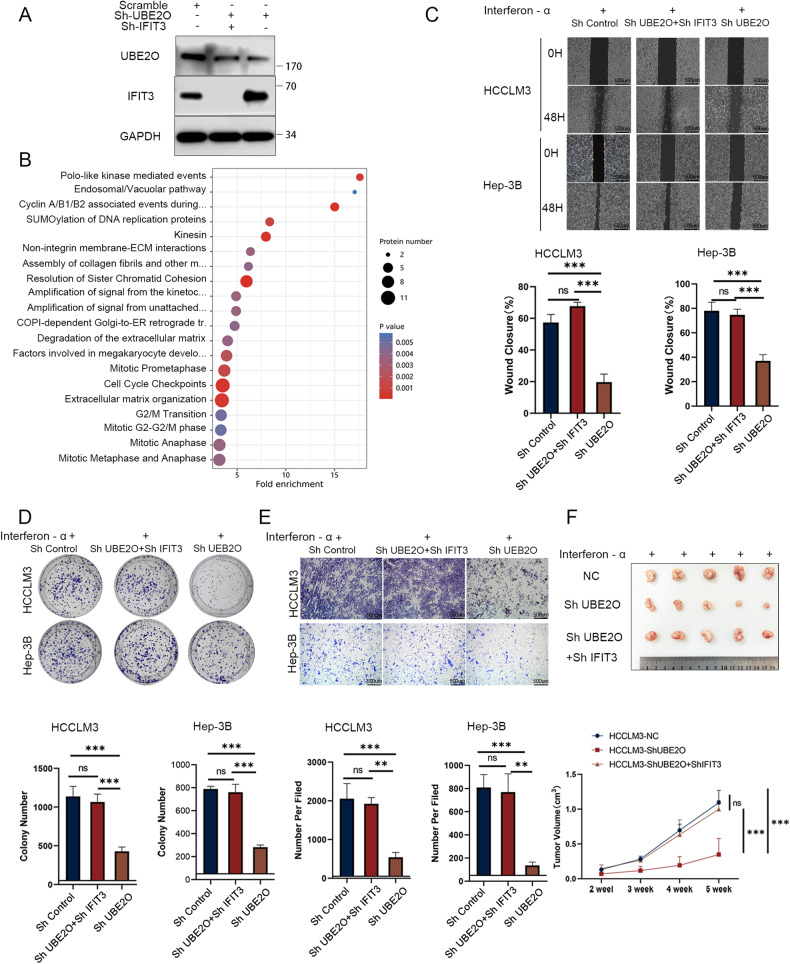
IFIT3 reverses UBE2O knockdown-mediated interferon-α treatment potentiation. Three kinds of cells were generated by transduction with the control, Sh-UBE2O, and Sh-IFIT3 lentiviral vectors, as shown in Fig. 6A. **A** Validation of the knockdown efficiency in the stably transduced cell lines. **B** Proteomic analysis was performed in the control and Sh-UBE2O+Sh-IFIT3 groups. The results showed that UBE2O no longer negatively regulated the interferon pathway after knockdown of IFIT3. Wound healing assays (**C**) colony formation assays (**D**) and migration assays (**E**) were used to verify the effect of the knockdown of UBE2O alone and simultaneous knockdown of UBE2O and IFIT3 on interferon sensitivity. Each set of experiments was performed with the same interferon concentration. The data are expressed as the mean ± SD of three independent experiments. *p < 0.05, **p < 0.01, *** *p* < 0.001, ns, nonsignificant. Ordinary one-way ANOVA with multiple comparisons testing was used to examine the statistical significance of differences among the three independent groups. **F** Stably transfected HCCLM3 cells from the control group, the UBE2O knockdown group, and the UBE2O and IFIT3 simultaneous knockdown group were injected into the inguinal region of mice, which were treated with the same dose of interferon, and the tumor volumes were calculated periodically (*n* = 5 mice/group). Ordinary one-way ANOVA with multiple comparisons testing was used for statistical analysis. ***p < 0.001, ns, nonsignificant.

We established stable transfectants of HCCLM3, Hep-3B, and Huh-7 cells with three different genotypes: control, UBE2O knockdown only, and simultaneous knockdown of UBE2O and IFIT3 (Fig. 6A). We utilized shIFIT3 to inhibit the increase in the IFIT3 level induced by ShUBE2O-mediated knockdown and performed proteomic analysis. The results showed that after IFIT3 knockdown, UBE2O did not affect interferon signaling (Fig. 6B). After downregulation of UBE2O, HCC cells exhibited an improved response to interferon-α, resulting in decreased cell proliferation and migration. However, the response to interferon-α remained unchanged when UBE2O and IFIT3 were simultaneously knocked down (Fig. 6C–E). Furthermore, simultaneous knockdown of IFIT3 and UBE2O did not significantly affect the subcutaneous tumor volume compared with that in the control group; however, knockdown of UBE2O alone resulted in a significant reduction in tumor volume (Fig. 6F) (*p* < 0.001). For interferon-α-resistant cells, knockdown of UBE2O was able to significantly increase the efficacy of interferon-α, and this effect was dependent on IFIT3 (Fig. S5A–D).

### ATO inhibits UBE2O to enhance the efficacy of interferon-α

**Fig. 7:**
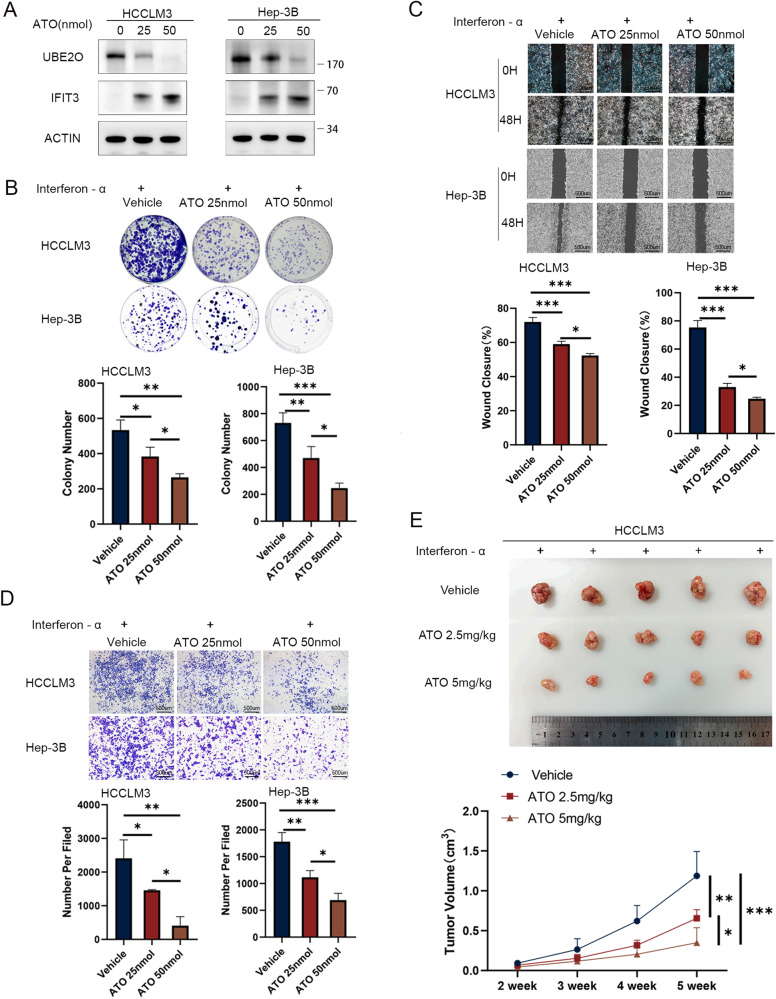
ATO improves the effect of interferon-α therapy by inhibiting UBE2O in HCC. Following treatment with interferon-α, cells were exposed to vehicle (0), 25 nmol of ATO, and 50 nmol of ATO for a duration of 4–8 h. Protein levels of UBE2O and IFIT3 were assessed via Western blot analysis (**A**). Cellular responses were evaluated using colony formation assays (**B**) wound healing assays (**C**) and migration assays (**D**). The images are from one representative experiment. The data are expressed as the mean ± SD of three independent experiments. *p < 0.05, **p < 0.01, ***p < 0.001. Ordinary one-way ANOVA with multiple comparisons testing was used to examine the statistical significance of differences among the three independent groups. (**E**) Three million HCCLM3 cells were implanted subcutaneously into nude mice. After 2 weeks, mice in the experimental and control groups were treated with interferon-α (5 × 10^6^ U/kg per day) combined with different concentrations of ATO (2.5 mg/kg or 5 mg/kg, daily), and tumor volumes were calculated periodically. Ordinary one-way ANOVA with multiple comparisons testing was used for statistical analysis. *p < 0.05, **p < 0.01, ***p < 0.001.

ATO is a recognized inhibitor of UBE2O [20]. After ATO therapy, the protein level of UBE2O decreased, while that of IFIT3 increased (Fig. 7A, and Fig. S10). When interferon-α was combined with two different concentrations of ATO, it was found that ATO enhanced the therapeutic effect of interferon-α by inhibiting the proliferation and migration of HCC cells. Higher concentrations of ATO were more effective than lower concentrations (all *p* < 0.05) (Fig. 7B–D). In the subcutaneous tumor xenograft model, the ATO intraperitoneal injection group exhibited a significant reduction in tumor volume (*p* < 0.05), with high-dose ATO treatment proving superior to low-dose treatment (*p* < 0.05) (Fig. 7E). Furthermore, the combination of ATO and interferon-α had a more pronounced effect on liver tumor cells than treatment with ATO alone. Higher concentrations of ATO were more effective in inhibiting UBE2O and elevating IFIT3 (Fig. S7A–D and Fig. S10).

## Discussion

The ubiquitin‒proteasome system remains a crucial target for novel anticancer drugs and chemosensitizers, and targeting this system is an important strategy to combat drug resistance in malignancies [11]. Based on analysis of TCGA and iUUCD data [28], UBE2O was identified. Ubiquitin ligases exert biological effects mainly by marking their substrates for degradation. Previous studies reported that in other tumor types, UBE2O mediates the degradation of AMPKα2 and TRAF6, thus affecting the AMPK and NFKB pathways, respectively, as well as EMT, DNA repair, and lipid metabolism [18, 25, 27, 33, 34]. Our data analyses and experiments did not reveal any degradation or binding of the aforementioned substrates in HCC. Therefore, we silenced UBE2O and conducted proteomic analysis to identify upregulated proteins. The results of the proteomic analysis suggested a negative regulatory relationship between UBE2O and both the overall interferon signaling pathway and the interferon α/β signaling pathway. We utilized mass spectrometry to identify IFT3, BST2, and TRIM21 as proteins that both bind to and are regulated by UBE2O. Additionally, we employed bioinformatics analysis to investigate the associations between UBE2O and these three proteins. The HPA database indicated that UBE2O exhibited protein expression patterns opposite those of IFIT3 in both liver tissue and hepatocellular carcinoma tissue, although its expression was positively correlated with that of BST2 and TRIM21. Survival analysis based on TCGA data revealed that low IFIT3 expression was associated with poor prognosis, while low IFIT3 expression combined with high UBE2O expression resulted in the worst prognosis. This negative correlation between UBE2O and IFIT3 protein levels was also confirmed in cell lines and tissue samples.

UBE2O acts as an E3 ubiquitin ligase, and it is reasonable to speculate that IFIT3 is a substrate of UBE2O in HCC. Our research suggests that IFIT3 has tumor-suppressive effects, while UBE2O acts as an oncogene. The results of TCGA data analysis supported our finding that patients with high expression of UBE2O and low expression of IFIT3 have the worst prognosis. Interferon-stimulated genes (ISGs) are essential components of the innate immune system [35]. IFIT3 is an interferon-induced protein and an inhibitor of cell migration, cell proliferation, apoptosis, and signal transduction. In a large multicenter study, patients with liver cancer were found to have low expression of IFIT1, IFIT2, and IFIT3. Only patients with high IFIT3 expression had significantly increased overall survival times after interferon-α treatment. Mechanistically, IFIT3 enhances IFN effector signaling by promoting the binding and nuclear translocation of STAT1 and STAT2 [10]. In our study, the results of both the in vitro and in vivo functional assays indicated that knockdown of UBE2O resulted in an increase in IFIT3 expression, leading to enhanced therapeutic efficacy of interferon-α. Furthermore, the results of the rescue experiments demonstrated that inhibition of IFIT3 reversed the potentiating effect of UBE2O knockdown on interferon-α treatment. Chikhalya and Xu focused on the downstream mechanism of IFIT3, and their findings corroborated the results of our study [36–38]. The K48-linked Ub chain is the main signal for proteasomal degradation, and in vivo, proteins with K63-linked ubiquitination are not degraded [39]. An in vivo ubiquitination assay was utilized to show that K236 of IFIT3 was the ubiquitination site by combining predictions from two databases and data from IP experiments. In summary, IFIT3 is degraded by UBE2O via K48-linked ubiquitination, and K236 is the site in IFIT3 for UBE2O-mediated ubiquitination.

ATO is an inhibitor of UBE2O [34]. ATO is a small molecule drug effective against hematological and solid cancers [40]. When taken with other medications, ATO has a potent inhibitory effect on multidrug resistance in cancer cells [41]. In vivo studies demonstrated that interferon-α + ATO is more effective than interferon-α alone in targeting Jak2V617F disease-initiating cells primarily through the action of PML [42]. Despite not being a first-line treatment, the use of ATO in combination with other drugs for tumor treatment has been widely reported [40–42]. In our study, UBE2O expression was inhibited and IFIT3 expression was elevated after ATO treatment, which enhanced the interferon-α effector response. In summary, UBE2O reduces the effectiveness of interferon-α by degrading IFIT3 in hepatocellular carcinoma.

### Reporting summary

Further information on research design is available in the Nature Research Reporting Summary linked to this article.

### Data Sharing Statement

The mass spectrometry proteomics data have been deposited to the ProteomeXchange Consortium with the dataset identifiers PXD039704 and PXD039711.

### Supplementary information

Supplementary Figures

Supplemental Figure and table legends

Original Data File

Supplementary Table 1

Supplementary Table 2

Supplementary Table 3

Supplementary Table 4

Supplementary Table 5

Supplementary Table 6

Supplementary Table 7

Supplementary Table 8

Supplementary Table 9

Reporting Summary
