# Supplementary material for: UBE2O reduces the effectiveness of interferon-α via degradation of IFIT3 in hepatocellular carcinoma
Source: Cell Death Dis. 2023 Dec 21;14(12):854. doi: 10.1038/s41419-023-06369-9 (PMC10740027; doi:10.1038/s41419-023-06369-9)

Figure S1

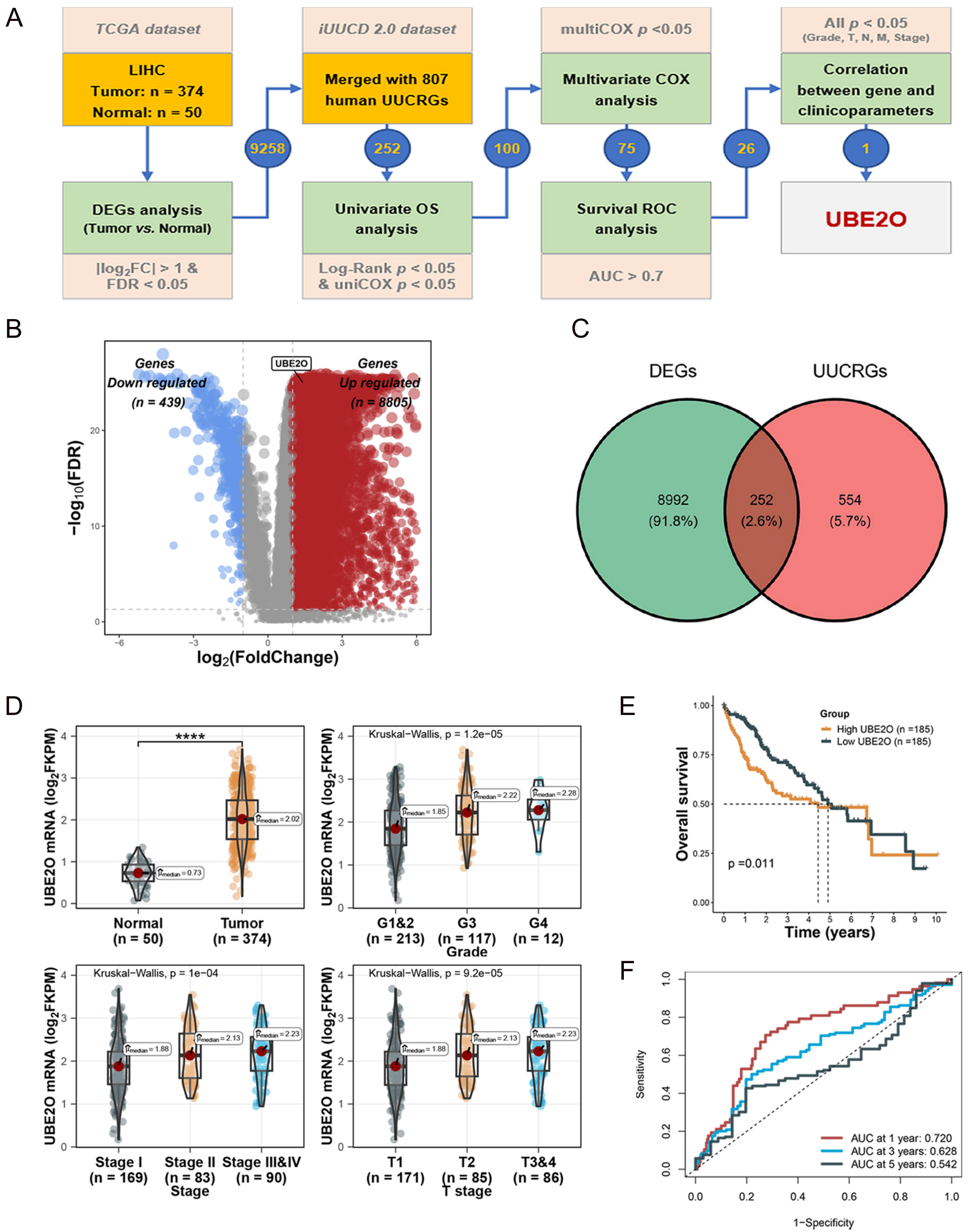
A
TCGA dataset
LIHC
Tumor: n = 374
Normal: n = 50
DEGs analysis
(Tumor vs. Normal)
|log2FC| > 1 &
FDR < 0.05
9258
iUUCD 2.0 dataset
Merged with 807
human UUCRGs
252
Univariate OS
analysis
Log-Rank p < 0.05
& uniCOX p < 0.05
100
multiCOX p <0.05
Multivariate COX
analysis
75
Survival ROC
analysis
AUC > 0.7
26
All p < 0.05
(Grade, T, N, M, Stage)
Correlation
between gene and
clinicoparameters
1
UBE2O
B
Genes
Down regulated
(n = 439)
UBE2O
Genes
Up regulated
(n = 8805)
-log10(FDR)
log2(FoldChange)
C
DEGs
UUCRGs
8992
(91.8%)
252
(2.6%)
554
(5.7%)
D
UBE2O mRNA (log2FKPM)
****
Normal
(n = 50)
Tumor
(n = 374)
Kruskal-Wallis, p = 1.2e-05
G1&2
(n = 213)
G3
(n = 117)
G4
(n = 12)
Grade
Kruskal-Wallis, p = 1e-04
Stage I
(n = 169)
Stage II
(n = 83)
Stage III&IV
(n = 90)
Stage
Kruskal-Wallis, p = 9.2e-05
T1
(n = 171)
T2
(n = 85)
T3&4
(n = 86)
T stage
E
Group
High UBE2O (n =185)
Low UBE2O (n =185)
Overall survival
p =0.011
Time (years)
F
Sensitivity
1-Specificity
AUC at 1 year: 0.720
AUC at 3 years: 0.628
AUC at 5 years: 0.542

Figure S2

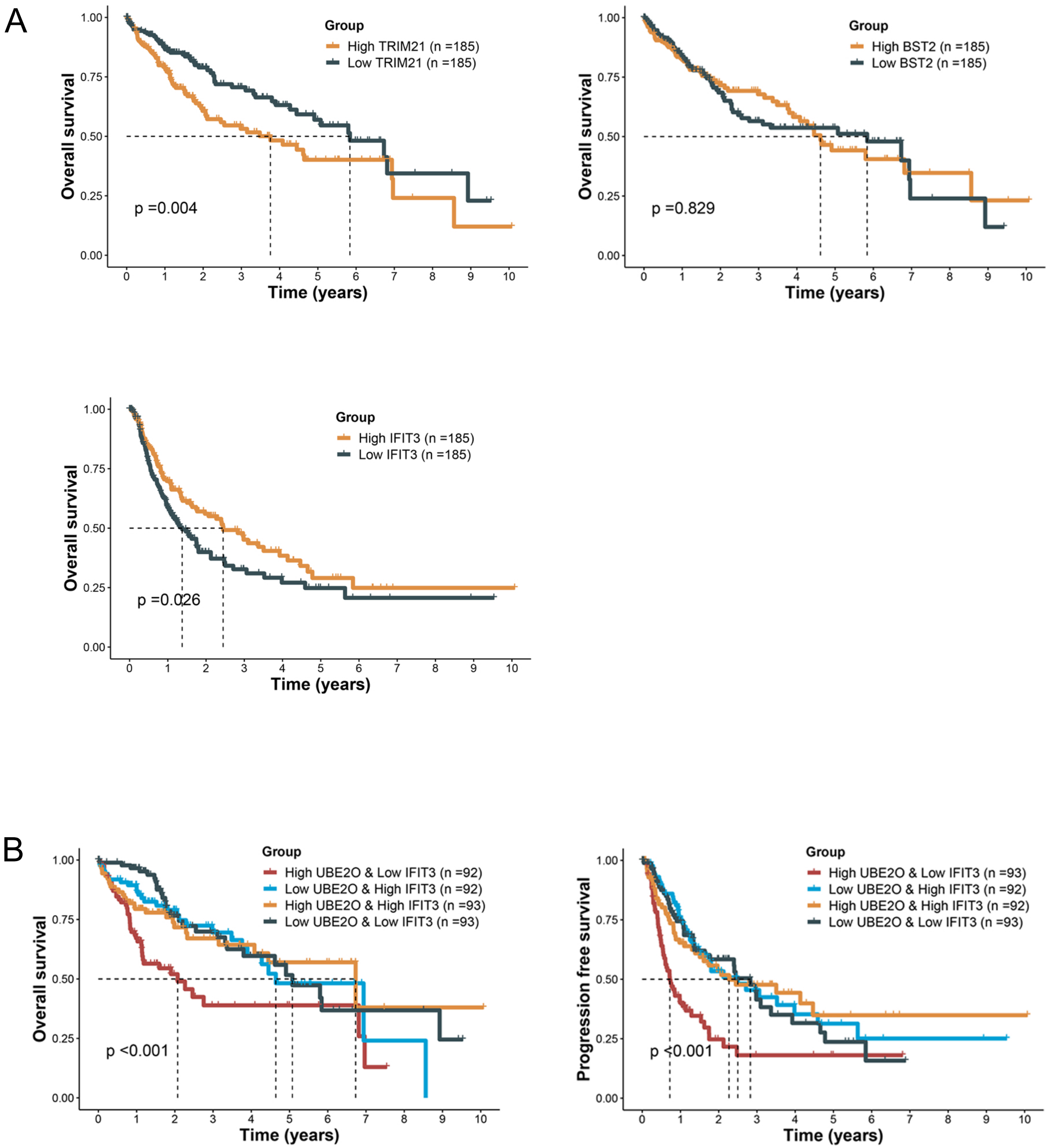

A
Group
High TRIM21 (n =185)
Low TRIM21 (n =185)
p =0.004
Overall survival
Time (years)
Group
High BST2 (n =185)
Low BST2 (n =185)
p =0.829
Overall survival
Time (years)
Group
High IFIT3 (n =185)
Low IFIT3 (n =185)
p =0.026
Overall survival
Time (years)
B
Group
High UBE2O & Low IFIT3 (n =92)
Low UBE2O & High IFIT3 (n =92)
High UBE2O & High IFIT3 (n =93)
Low UBE2O & Low IFIT3 (n =93)
p <0.001
Overall survival
Time (years)
Group
High UBE2O & Low IFIT3 (n =93)
Low UBE2O & High IFIT3 (n =92)
High UBE2O & High IFIT3 (n =92)
Low UBE2O & Low IFIT3 (n =93)
p <0.001
Progression free survival
Time (years)

Figure S3

A

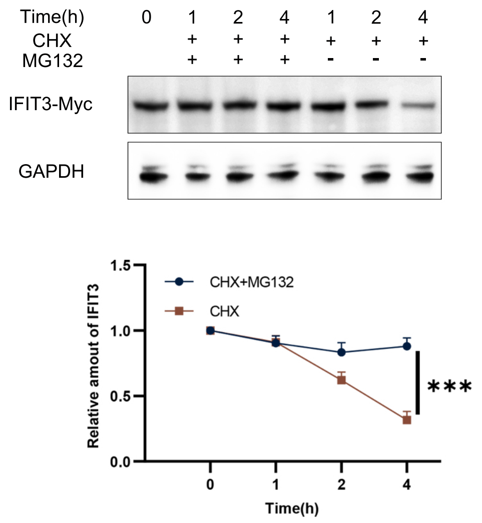

B

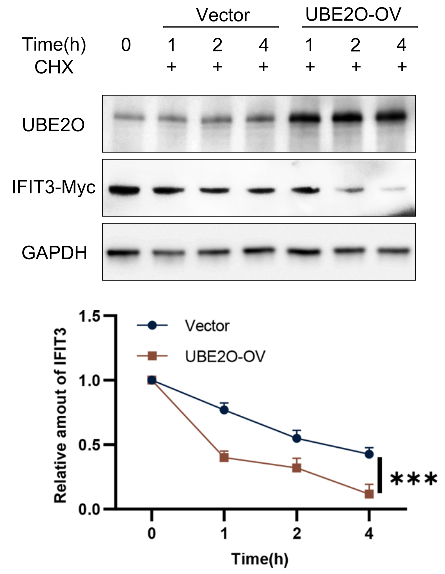

C

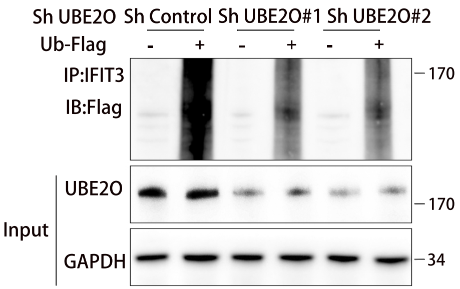

D

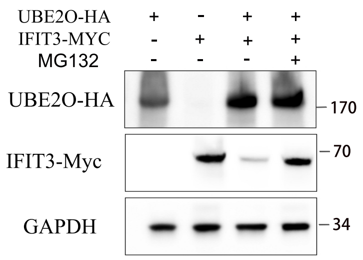

F

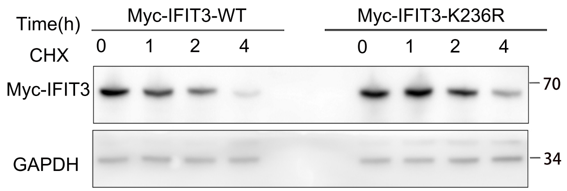

E

GEQFVEEALEKSPCQTDVLRSAAKFY----- Homo sapiens
GEQLVEEALEKAPCQTDVLRSAAKFY----- Pongo abelii
AERLVEDALEKGPNQTDVLQKAAQFY---- Rattus norvegicus
GEQFVKEALEKAPCQTDVLRSAANFY----- Oryctolagus cuniculus
GEQFVEEALEKAPCQTDVLRSAAKFY----- Pan troglodytes

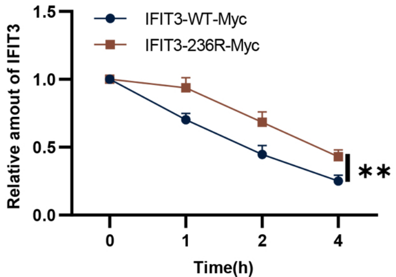

Figure S4

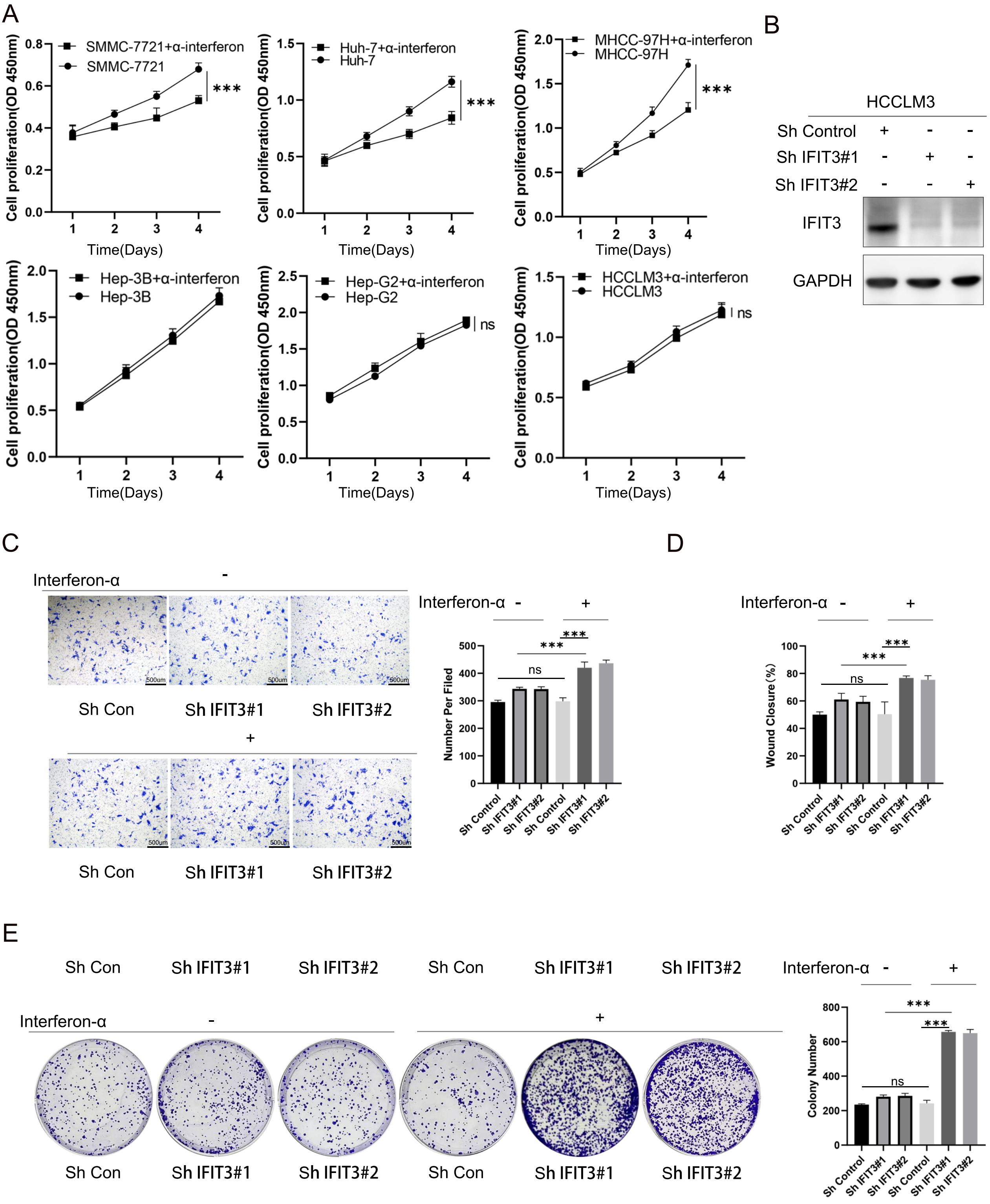
A
SMMC-7721+α-interferon
SMMC-7721
Huh-7+α-interferon
Huh-7
MHCC-97H+α-interferon
MHCC-97H
Hep-3B+α-interferon
Hep-3B
Hep-G2+α-interferon
Hep-G2
HCCLM3+α-interferon
HCCLM3
Cell proliferation(OD 450nm)
Time(Days)
***
ns
B
HCCLM3
Sh Control + - -
Sh IFIT3#1 - + -
Sh IFIT3#2 - - +
IFIT3
GAPDH
C
Interferon-α - +
Sh Con
Sh IFIT3#1
Sh IFIT3#2
500um
Number Per Filed
Sh Control
Sh IFIT3#1
Sh IFIT3#2
D
Wound Closure (%)
E
Colony Number

Figure S5

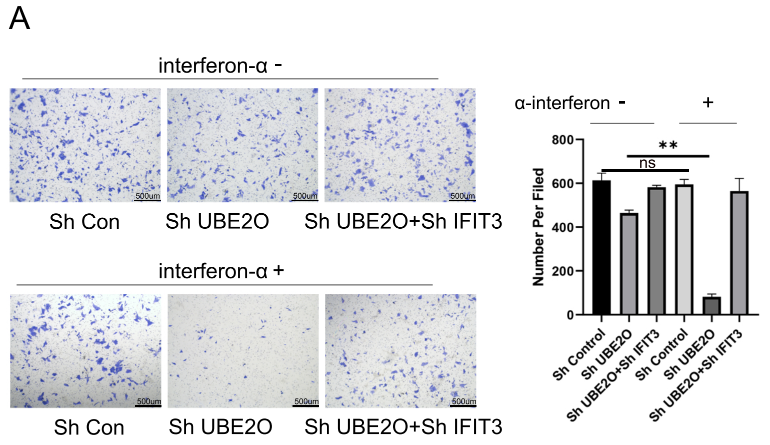
A
interferon-α -
Sh Con
Sh UBE2O
Sh UBE2O+Sh IFIT3
interferon-α +
Sh Con
Sh UBE2O
Sh UBE2O+Sh IFIT3
α-interferon - +
Number Per Filed
ns
**
Sh Control
Sh UBE2O
Sh UBE2O+Sh IFIT3

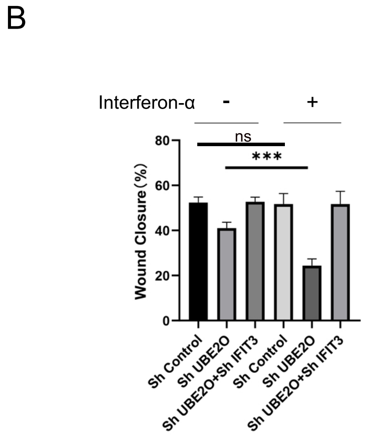
B
Interferon-α - +
Wound Closure (%)
ns
***
Sh Control
Sh UBE2O
Sh UBE2O+Sh IFIT3

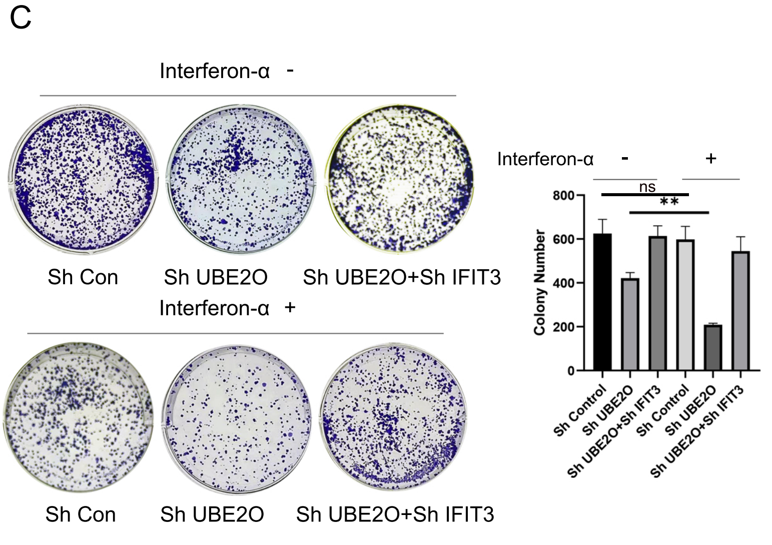
C
Interferon-α -
Sh Con
Sh UBE2O
Sh UBE2O+Sh IFIT3
Interferon-α +
Interferon-α - +
Colony Number
ns
**
Sh Control
Sh UBE2O
Sh UBE2O+Sh IFIT3

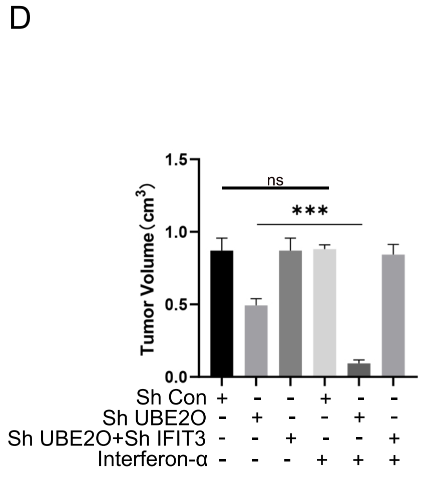
D
Tumor Volume (cm³)
ns
***
Sh Con + - - + - -
Sh UBE2O - + - - + -
Sh UBE2O+Sh IFIT3 - - + - - +
Interferon-α - - - + + +

Figure S6

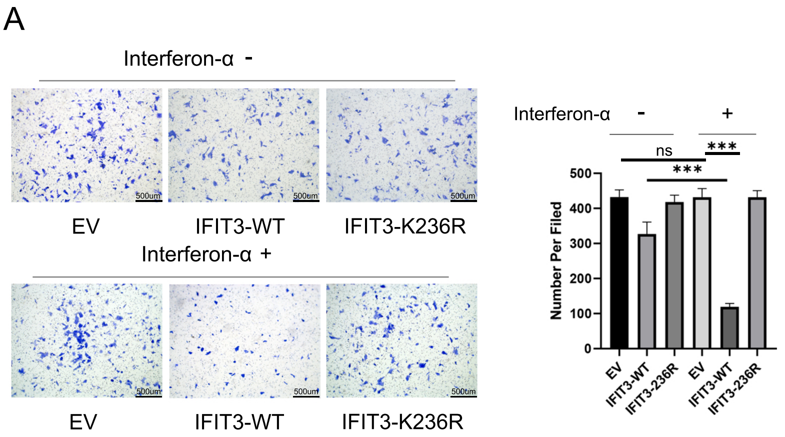
A
Interferon-α -
500um
EV
IFIT3-WT
IFIT3-K236R
Interferon-α +
Interferon-α
-
+
ns
***
Number Per Filed
500
400
300
200
100
0
IFIT3-236R

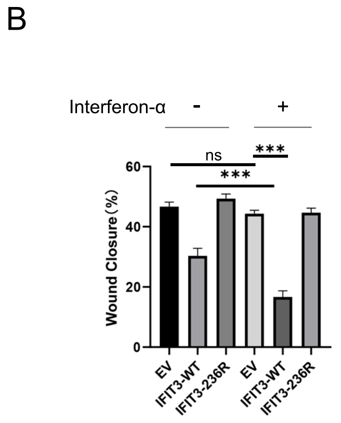
B
Interferon-α
-
+
ns
***
Wound Closure (%)
60
40
20
0
EV
IFIT3-WT
IFIT3-236R

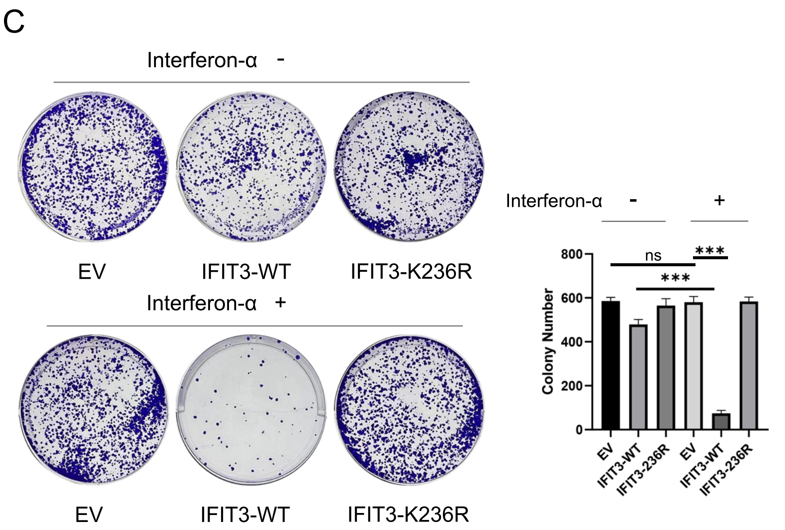
C
Interferon-α -
EV
IFIT3-WT
IFIT3-K236R
Interferon-α +
Interferon-α
ns
***
Colony Number
800
600
400
200
0
IFIT3-236R

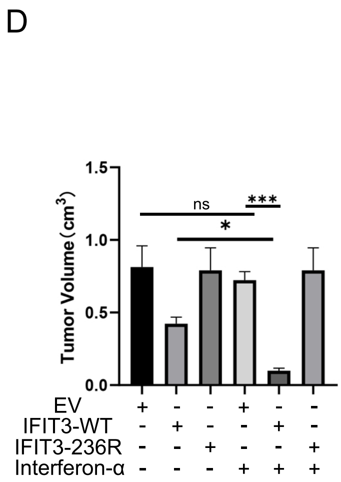
D
ns
***
*
Tumor Volume (cm3)
1.5
1.0
0.5
0.0
EV + - - + - -
IFIT3-WT - + - - + -
IFIT3-236R - - + - - +
Interferon-α - - - + + +

Figure S7

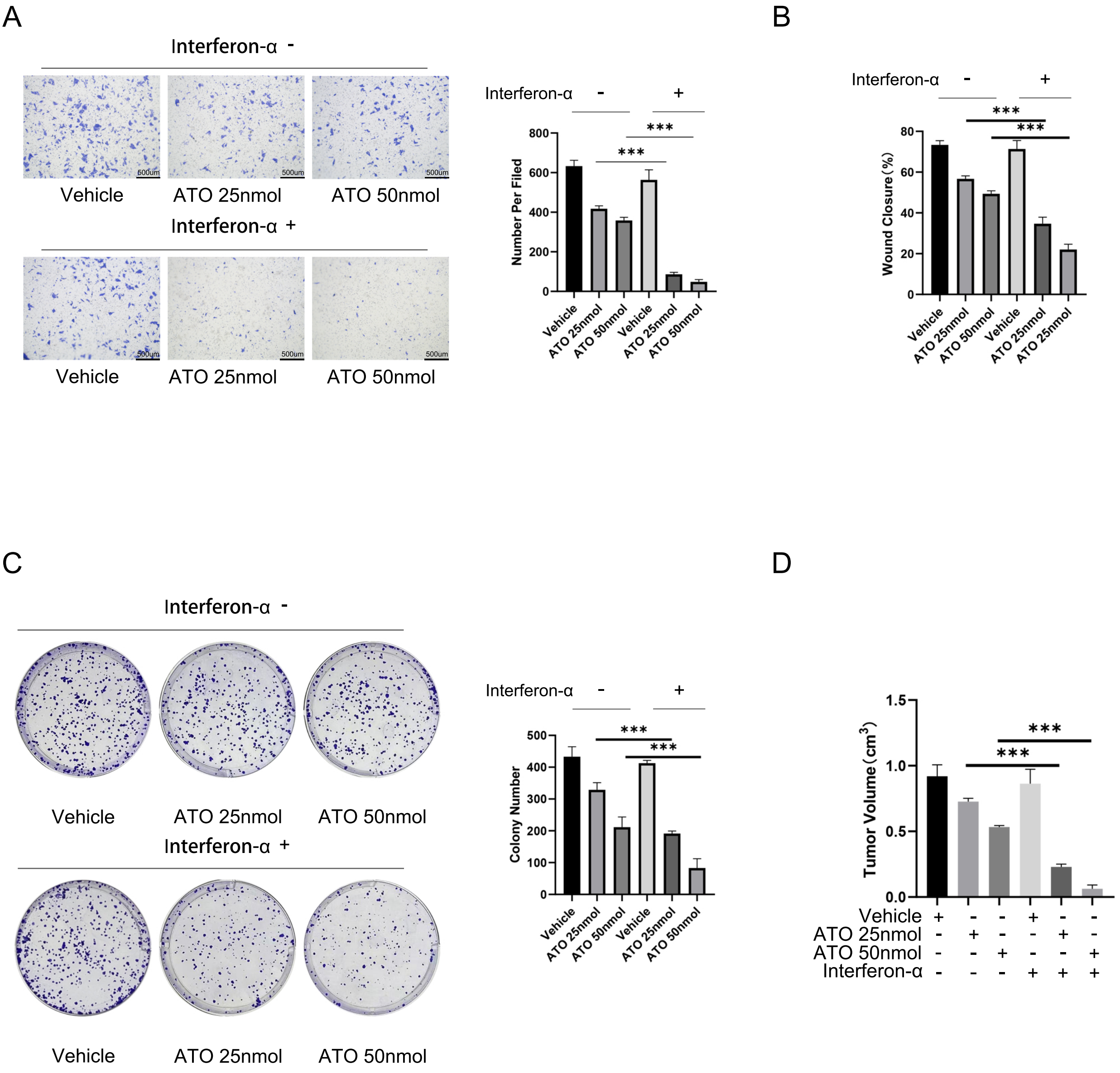

A
Interferon-α -
500um
Vehicle
ATO 25nmol
ATO 50nmol
Interferon-α +
Vehicle
ATO 25nmol
ATO 50nmol
Interferon-α - +
***
***
Number Per Filed
800
600
400
200
0
Vehicle
ATO 25nmol
ATO 50nmol
Vehicle
ATO 25nmol
ATO 50nmol
B
Interferon-α - +
***
***
Wound Closure (%)
80
60
40
20
0
Vehicle
ATO 25nmol
ATO 50nmol
Vehicle
ATO 25nmol
ATO 25nmol
C
Interferon-α -
Vehicle
ATO 25nmol
ATO 50nmol
Interferon-α +
Vehicle
ATO 25nmol
ATO 50nmol
Interferon-α - +
***
***
Colony Number
500
400
300
200
100
0
Vehicle
ATO 25nmol
ATO 50nmol
Vehicle
ATO 25nmol
ATO 50nmol
D
***
***
Tumor Volume (cm3)
1.5
1.0
0.5
0.0
Vehicle + - - + - -
ATO 25nmol - + - - + -
ATO 50nmol - - + - - +
Interferon-α - - - + + +

Figure S8

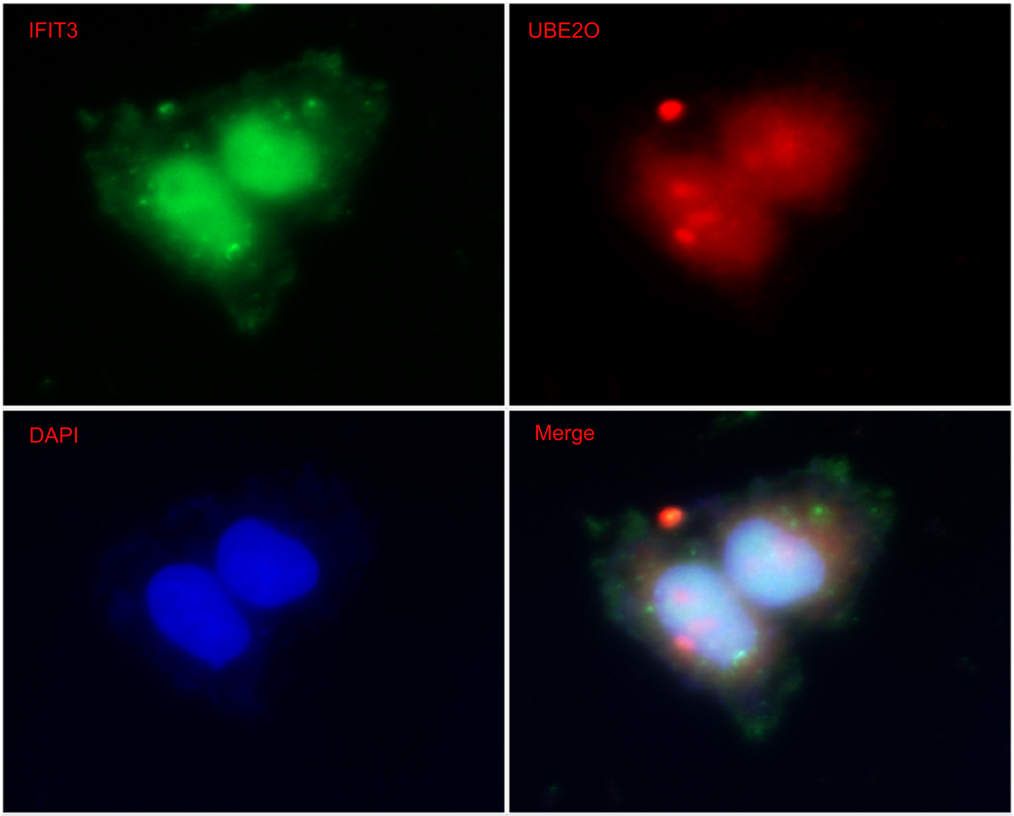

Figure S9

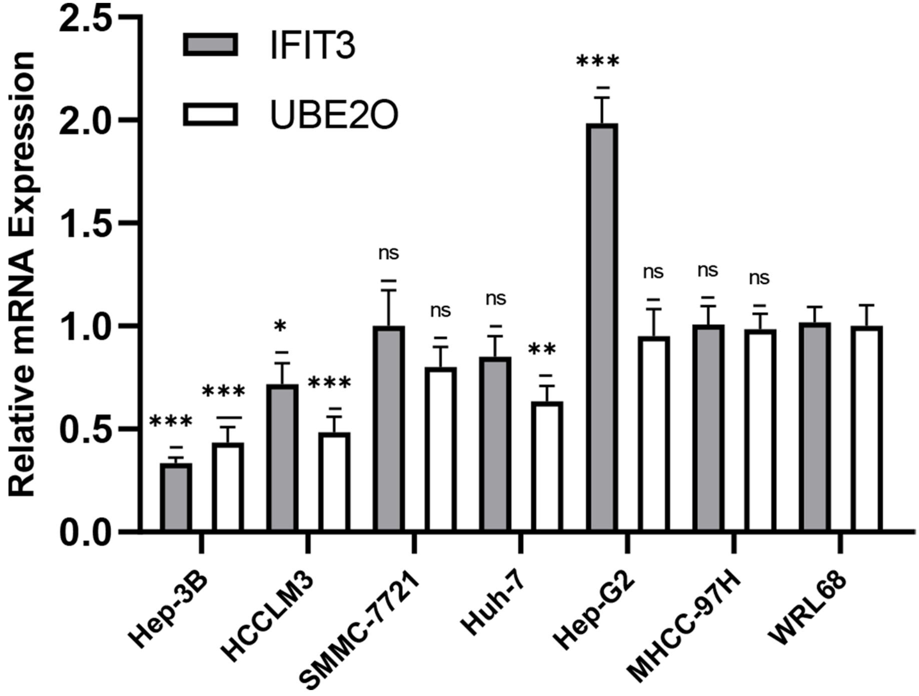
2.5
2.0
1.5
1.0
0.5
0.0
Relative mRNA Expression
IFIT3
UBE2O
***
***
*
***
ns
ns
ns
**
***
ns
ns
ns
Hep-3B
HCCLM3
SMMC-7721
Huh-7
Hep-G2
MHCC-97H
WRL68

Figure S10

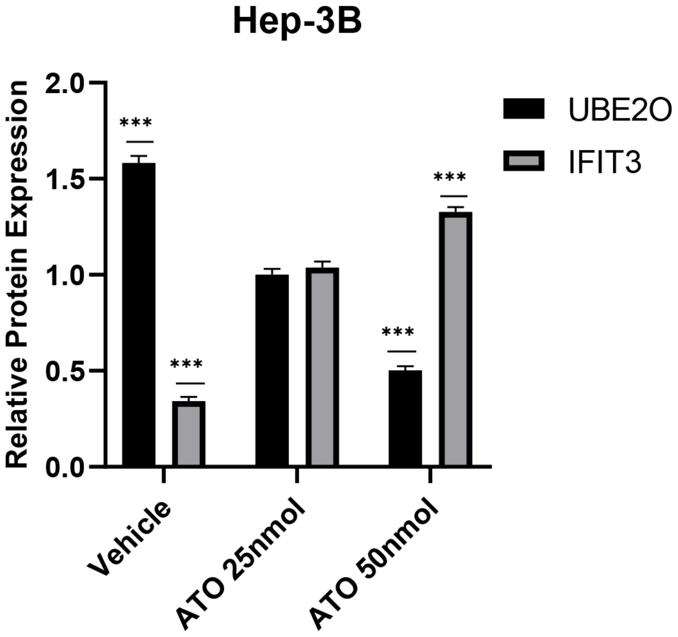
Hep-3B
Relative Protein Expression
2.0
1.5
1.0
0.5
0.0
***
***
***
***
UBE2O
IFIT3
Vehicle
ATO 25nmol
ATO 50nmol

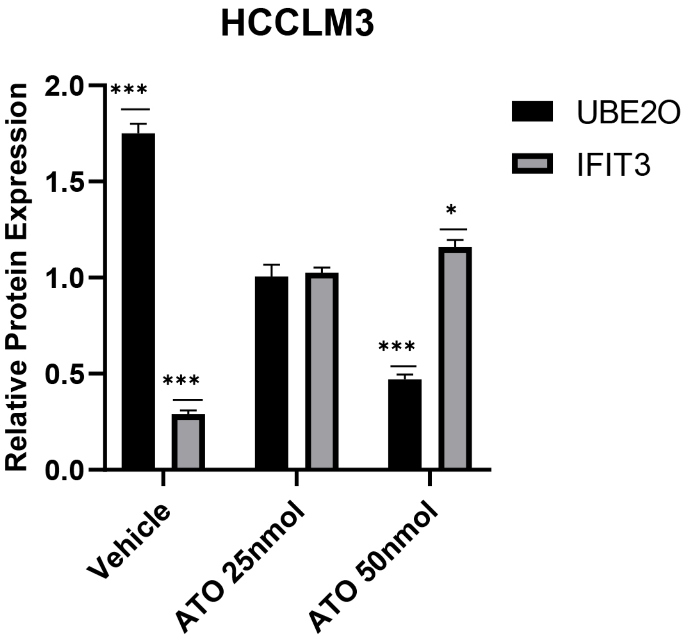
HCCLM3
Relative Protein Expression
2.0
1.5
1.0
0.5
0.0
***
***
***
*
UBE2O
IFIT3
Vehicle
ATO 25nmol
ATO 50nmol

Figure S11

A

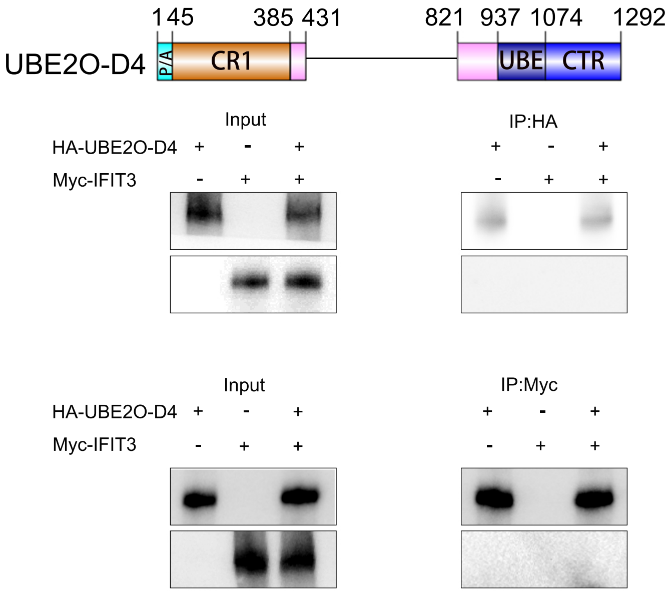

B

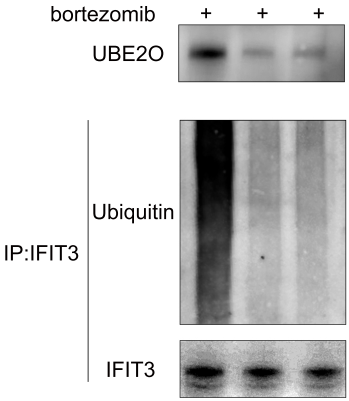

C

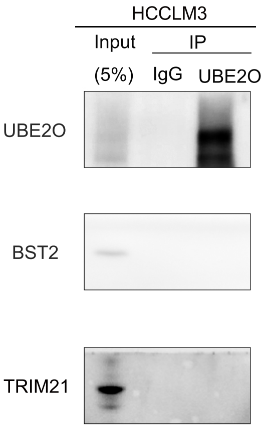

Supplement: Supplementary file 1 — Supplementary Figures [file 41419_2023_6369_MOESM1_ESM.pdf]
