# Supplementary material for: UBE2O reduces the effectiveness of interferon-α via degradation of IFIT3 in hepatocellular carcinoma
Source: Cell Death Dis. 2023 Dec 21;14(12):854. doi: 10.1038/s41419-023-06369-9 (PMC10740027; doi:10.1038/s41419-023-06369-9)
Supplement: Supplementary file 2 — Supplemental Figure and table legends [file 41419_2023_6369_MOESM2_ESM.docx]

Figure S1: **Among UUCRGs, UBE2O is a promising therapeutic target in hepatocellular carcinoma.** (A) Schematic diagram of the screening process for UUCRGs. (B) Differentially expressed genes (DEGs) in HCC *vs*. normal tissues based on the TCGA LIHC dataset. (C) Venn diagrams showing a total of 252 common genes overlapping between UUCRGs and the identified DEGs. (C) In the TCGA database, the mRNA expression level of UBE2O was significantly higher in hepatocellular carcinoma tissue than in normal liver tissue. (D) In the TCGA database, the mRNA expression level of UBE2O in HCC tissue was analyzed, and correlation analysis between the genes and five major clinicopathological parameters of the patients was performed. (E) Kaplan–Meier analysis of survival based on UBE2O expression in the LIHC cohort. (F) Time-dependent ROC analyses based on UBE2O expression.

Figure S2: **Survival analysis based on the expression of the involved genes in HCC.** (A) OS analysis based on IFIT3, BST2, and TRIM21 expression in the TCGA LIHC dataset. (B) The corresponding OS and PFS curves of patients with high UBE2O expression combined with low IFIT3 expression.

Figure S3: **UBE2O mediates the ubiquitination and degradation of IFIT3.** (A) The MYC-IFIT3 plasmid was transfected into 293T cells treated with or without MG132 as indicated, and the corresponding protein expression levels were subsequently analyzed by Western blotting. (B) UBE2O was overexpressed in 293T cells, which were subsequently transfected with the IFIT3 plasmid. These cells were harvested at the indicated time points after CHX treatment for Western blot analysis using an anti-IFIT3 antibody. (C) 293T cells were transfected with sh-UBE2O or sh-Control as indicated, followed by treatment with MG132 (10 μM, 6-8 h) prior to lysis. The lysates were subjected to immunoprecipitation with an anti-IFIT3 antibody and subsequent immunoblot analysis with an anti-Flag antibody. (D) UBE2O and IFIT3 plasmids, each containing different tags, were cotransfected into 293T cells. The resulting changes in IFIT3 protein levels were then compared to those observed in the MG132 treatment group. (E) Alignment of the amino acid sequences containing K236 in IFIT3 from various species. (F) The IFIT3-Myc-WT or IFIT3-Myc-K236R plasmid was transfected into 293T cells, followed by treatment with CHX. The cells were harvested at a predetermined time point and subjected to analysis of IFIT3 protein expression.

**Figure S4. Inhibition of IFIT3 reduces the efficacy of interferon-α therapy.** (A) CCK8 analysis and cell growth curves were conducted on liver tumor cells treated with normal saline or interferon-α. (B) Lentivirus was transfected into HCCLM3 cells to knock down IFIT3, and the effectiveness was assessed using Western blot analysis. Migration (C), wound healing (D), and colony formation assays (E) were performed following IFIT3 gene knockdown in combination with normal saline or interferon-α treatment. The images are from one representative experiment. The data are expressed as the mean ± SD of three independent experiments. *p < 0.05, **p < 0.01, ***p < 0.001. Ordinary one-way ANOVA with multiple comparisons testing was used for statistical analysis.

**Figure S5. The inhibitory effect of UBE2O on interferon-α efficacy is contingent upon the presence of IFIT3.** Three separate experimental groups were established: a control group, a group with UBE2O knockdown alone, and a group with both UBE2O and IFIT3 knockdown. Migration (A), wound healing (B), and colony formation (C) were assessed in the absence and presence of interferon-α. The data are expressed as the mean ± SD of three independent experiments. *p < 0.05, **p < 0.01, ***p < 0.001. Ordinary one-way ANOVA with multiple comparisons testing was used for statistical analysis. (D), Three distinct cell types (Sh Control, Sh UBE2O, Sh UBE2O+Sh IFIT3) were generated through stable transfection in HCCLM3 cells. Subsequently, equal quantities of these cell types were injected into the groin region of nude mice, with one group receiving interferon-α treatment and the other serving as a saline control. Regular monitoring was conducted to assess tumor volume. Ordinary one-way ANOVA with multiple comparisons testing was used for statistical analysis.

**Figure S6.** **The K236 site on IFIT3 is critical for interferon-α efficacy.** The stably transfected HCCLM3 cells in the control group, IFIT3-WT group, and IFIT3-236R group were constructed. (A) Migration, (B) wound healing, and (C) clonal formation were detected in the absence and presence of interferon-α. The data are expressed as the mean ± SD of three independent experiments. *p < 0.05, **p < 0.01, ***p < 0.001. Ordinary one-way ANOVA with multiple comparisons testing was used for statistical analysis. (D) Stable transfection was performed in HCCLM3 cells to generate three types of cells (EV, IFIT3-WT, IFIT3-236R). Subsequently, equal numbers of these cell types were injected into the groin region of nude mice, with one group receiving interferon-α treatment and the other serving as a saline control. And tumor volume was monitored at regular intervals. Ordinary one-way ANOVA with multiple comparisons testing was used for statistical analysis.

**Figure S7. ATO enhances the efficacy of interferon-α in HCC.** HCCLM3 cells were treated with three concentrations of ATO (0, 25 nmol, and 50 nmol) either alone or in combination with interferon-α. Subsequently, migration assays (A), wound healing assays (B), and colony formation tests (C) were conducted. The data are expressed as the mean ± SD of three independent experiments. *p < 0.05, **p < 0.01, ***p < 0.001. Ordinary one-way ANOVA with multiple comparisons testing was used for statistical analysis. (D) The groin of nude mice was implanted with 300 HCCLM3 cells. Subsequently, they were subjected to treatment with varying concentrations of ATO (0, 2.5 mg/kg, or 5mg/kg) in combination with saline or different concentrations of ATO in combination with interferon (5×106 U/kg/day), and the tumor volume was periodically measured. Ordinary one-way ANOVA with multiple comparisons testing was used for statistical analysis.

**Figure S8,** **UBE2O and IFIT3 interactions.** In HCCLM3 cells, immunofluorescence was used to demonstrate the localization of UBE2O in relation to IFIT3 in the cells.

**Figure S9. RNA expression of IFIT3 and UBE2O in liver tumor cells and normal liver cells.**

**Figure S10. Statistical analysis of UBE2O and IFIT3 protein expression levels after treatment of hepatocellular carcinoma cells with different concentrations of ATO.**

**Figure S11. The E3 ligase UBE2O specifically targets IFIT3 for ubiquitination and degradation.** (A) 293T cells were transfected with UBE2O-D4 and IFIT3 plasmids, followed by immunoprecipitation using Myc-tag and HA-tag antibodies, respectively. (B) Sh Control and Sh UBE2O HCCLM3 cells were treated with bortezomib for 12 hours, followed by immunoprecipitation using IFIT3 antibodies. Protein samples were subjected to Western blot analysis and probed with ubiquitin antibodies. (C) An immunoprecipitation assay was performed in HCCLM3 cells using a UBE2O antibody, followed by Western blot analysis employing BST2 and TRIM21 antibodies.

Supplementary Table 1: **Differentially expressed genes (DEGs) in HCC *vs.* normal tissues based on the TCGA LIHC dataset.**

Supplementary Table 2: **Human ubiquitin and ubiquitin-like conjugation-related genes (UUCRGs, n=807) downloaded from the iUUCD2.0 database.**

Supplementary Table 3: **Expression matrix of the 252 genes overlapping between the DEGs and UUCRGs in the TCGA LIHC dataset.**

Supplementary Table 4: **Results of Kaplan‒Meier and univariate Cox regression analysis of overall survival based on the 252 overlapping genes.**

Supplementary Table 5: **Results of multivariate Cox regression analysis of overall survival based on the 252 overlapping genes (multiCOX p < 0.05 considered to indicate a statistically significant difference).**

Supplementary Table 6: **ROC analysis of survival data based on the 252 overlapping genes.**

Supplementary Table 7: **Correlation analysis between gene expression and clinicopathological parameters.**

Supplementary Table 8: **UBE2O-associated proteins identified by mass spectrometry.**

Supplementary Table 9: **Antibodies, primers, siRNA sequences, and shRNA sequences.**
