# Supplementary material for: UBE2O reduces the effectiveness of interferon-α via degradation of IFIT3 in hepatocellular carcinoma
Source: Cell Death Dis. 2023 Dec 21;14(12):854. doi: 10.1038/s41419-023-06369-9 (PMC10740027; doi:10.1038/s41419-023-06369-9)
Supplement: Supplementary file 12 — Supplementary Table 9 [file 41419_2023_6369_MOESM12_ESM.doc]

Supplementary Table 9: Antibodies, Primer, Si-RNA sequence, and shRNA sequences

| Antigens | Antibody information |
| --- | --- |
| UBE2O | Polyclonal Antibody (PA5-87583, AB_2804268) |
| IFIT3 | Polyclonal antibody (Cat No. 15201-1-AP, AB_2248738) |
| GAPDH | Monoclonal antibody (Cat No. 60004-1-Ig, AB_2107436) |
| Beta Actin | Monoclonal antibody (Cat No. 81115-1-RR, AB_2687938) |
| TRIM21 | Polyclonal antibody (Cat No. 12108-1-AP, AB_2209469) |
| HA tag | Monoclonal antibody (Cat No. 51064-2-AP, AB_2881490) |
| DYKDDDDK tag | Monoclonal antibody (Binds to FLAG® tag epitope) (Cat No. 66008-4-Ig, AB_2918475) |
| MYC tag | Polyclonal antibody (Cat No. 16286-1-AP, AB_11182162) |
| ubiquitin | Polyclonal antibody (Cat No. 10201-2-AP) |
| BST2 | Polyclonal antibody (Cat No: 13560-1-AP, AB_2067220) |

Primer RNA list

| Name | Species | Forward primer sequence | Reversed primer sequence |
| --- | --- | --- | --- |
| UBE2O | Ho | ACTAGAGGACCGTTCTGTGGT | TGACGGGATAGATGATGCAGTT |
| IFIT3 | Ho | TCAGAAGTCTAGTCACTTGGGG | ACACCTTCGCCCTTTCATTTC |
| GAPDH | Ho | GAGAAGTATGACAACAGCCTCAA | GCCATCACGCCACAGTTT |

Si-RNA sequence

| Name | Species | Forward | Reversed |
| --- | --- | --- | --- |
| Si-IFIT3-1 | Ho | ACGGCAAGCUGAAGAGUUATT | UAACUCUUCAGCUUGCCGUTT |
| Si-IFIT3-2 | Ho | GAUUGAAGCACUAAAGCAATT | UUGCUUUAGUGCUUCAAUCTT |
| Si-IFIT3-3 | Ho | CUGACAAGGAAGAGAUCAATT | UUGAUCUCUUCCUUGUCAGTT |

Short hairpin (Sh) RNA sequence

| Name | Sequence |
| --- | --- |
| Sh-Control | GATCCGTTCTCCGAACGTGTCACGTAATTCAAGAGATTACGTGACACGTTCGGAGAATTTTTTC  AATTGAAAAAATTCTCCGAACGTGTCACGTAATCTCTTGAATTACGTGACACGTTCGGAGAACG |
| Sh-UBE2O-1 | GATCCGTCGTCATCCGCATCGGCAATACTCGAGTATTGCCGATGCGGATGACGATTTTTTG  AATTCAAAAAATCGTCATCCGCATCGGCAATACTCGAGTATTGCCGATGCGGATGACGACG |
| Sh-UBE2O-2 | GATCCGATGTGAGTGTTTACGACATTGCTCGAGCAATGTCGTAAACACTCACATTTTTTTG |
|  | AATTCAAAAAAATGTGAGTGTTTACGACATTGCTCGAGCAATGTCGTAAACACTCACATCG |
| Sh-UBE2O-3 | GATCCGCGGGTCTCTTCTTCGATGATTCTCGAGAATCATCGAAGAAGAGACCCGTTTTTTG  AATTCAAAAAACGGGTCTCTTCTTCGATGATTCTCGAGAATCATCGAAGAAGAGACCCGCG |
